# Derivation of Injury-Responsive Dendritic Cells for Acute Brain Targeting and Therapeutic Protein Delivery in the Stroke-Injured Rat

**DOI:** 10.1371/journal.pone.0061789

**Published:** 2013-04-16

**Authors:** Nathan C. Manley, Javier R. Caso, Melissa G. Works, Andrew B. Cutler, Ilona Zemlyak, Guohua Sun, Carolina D. Munhoz, Sydney Chang, Shawn F. Sorrells, Florian V. Ermini, Johannes H. Decker, Anthony A. Bertrand, Klaus M. Dinkel, Gary K. Steinberg, Robert M. Sapolsky

**Affiliations:** 1 Department of Biology, Stanford University, Stanford, California, United States of America; 2 Department of Neurosurgery, Stanford University School of Medicine, Stanford, California, United States of America; 3 Stanford Stroke Center and Stanford Institute for Neuro-Innovation and Translational Neurosciences, Stanford University School of Medicine, Stanford, California, United States of America; 4 Department of Bioengineering, Stanford University School of Medicine, Stanford, California, United States of America; 5 Department of Pharmacology, Institute of Biomedical Sciences, University of São Paulo, Sao Paulo, Brazil; 6 Department of Neurology and Neurological Sciences, Stanford University School of Medicine, Stanford, California, United States of America; University of Münster, Germany

## Abstract

Research with experimental stroke models has identified a wide range of therapeutic proteins that can prevent the brain damage caused by this form of acute neurological injury. Despite this, we do not yet have safe and effective ways to deliver therapeutic proteins to the injured brain, and this remains a major obstacle for clinical translation. Current targeted strategies typically involve invasive neurosurgery, whereas systemic approaches produce the undesirable outcome of non-specific protein delivery to the entire brain, rather than solely to the injury site. As a potential way to address this, we developed a protein delivery system modeled after the endogenous immune cell response to brain injury. Using ex-vivo-engineered dendritic cells (DCs), we find that these cells can transiently home to brain injury in a rat model of stroke with both temporal and spatial selectivity. We present a standardized method to derive injury-responsive DCs from bone marrow and show that injury targeting is dependent on culture conditions that maintain an immature DC phenotype. Further, we find evidence that when loaded with therapeutic cargo, cultured DCs can suppress initial neuron death caused by an ischemic injury. These results demonstrate a non-invasive method to target ischemic brain injury and may ultimately provide a way to selectively deliver therapeutic compounds to the injured brain.

## Introduction

In recent decades, research has identified a wide range of drugs and other therapeutic agents that can suppress brain damage caused by an acute neurological injury, such as stroke, yet none of these neuroprotective compounds have translated to the clinic. A major obstacle has been effective delivery of compounds, ideally requiring a way to target the injured brain within an appropriate time window. Current methods for injury-specific delivery typically involve direct intraparenchymal infusions of either viral vectors or genetically-engineered cells [1], [2], raising significant safety concerns. In contrast, systemic delivery approaches, such as transgene-liposome conjugates [3] or therapeutic proteins fused to a BBB-specific antibody [4], allow non-invasive transport from the bloodstream to the brain parenchyma, but do so non-specifically so that the therapeutic agent is delivered throughout the body and to both injured and uninjured brain regions. Although these delivery strategies have proven effective in experimental injury models, clinical treatment of brain injury could benefit greatly from a non-invasive and highly specific delivery system for therapeutic compounds.

Interestingly, such injury targeting occurs endogenously, in the form of peripheral immune cells that localize to damaged tissue during a central nervous system (CNS) injury, such as stroke [5], [6]. Acting as recruitment signals for this immune response, cytokines and chemmoattractant chemokines produced at the injury site attract migrating peripheral immune cells, and local upregulation of adhesion molecules by vascular endothelial cells allows recruited cells to firmly bind to neurovascular endothelial cells present at the BBB, and in some cases, extravasate into the brain parenchyma [7], [8]. Peripheral immune cell recruitment includes an innate response by granulocytes, macrophages, monocytes and dendritic cells (DCs) within the first 24 hours as well as delayed homing of lymphocytes in the subsequent days post-injury [6]. From the perspective of therapeutic delivery, this immune response is an attractive system because it offers a way to target different therapeutic windows depending on the cell type used. Moreover, from the perspective of selectivity, this immune response provides an ideal model, as by definition, these cells are only recruited to areas of the brain where therapy is needed.

Indeed, recent studies have shown that endogenous peripheral immune cells can transport therapeutic proteins to the CNS. In a mouse Alzheimer's model, peripheral infusion of blood-derived immune cells resulted in their homing to CNS amyloid plaques, and prior loading of these cells with a secretable version of the A-beta cleaving enzyme, neprilysin, reduced amyloid plaque burden [9]. In a rat model of stroke, endogenous peripheral macrophages were shown to phagocytose transgene-loaded liposomes in vivo and deliver them to the infarct, and post-injury delivery of fibroblast growth factor 2 resulted in increased neurogenesis in the lesioned hemisphere [10]. These studies demonstrate that endogenous peripheral immune cells can target both chronic and acute CNS injuries and can have a therapeutic impact even when administered during the post-injury/recovery phase. However, an important remaining question is whether subtypes of immune cells can be used to target the initial injury phase, as such an approach could have particular benefit for acute CNS injuries. In addition, it is not known if injury-homing capacity is unique to cells generated in vivo. If instead, injury targeting can be achieved by cells derived ex vivo, this could provide an ideal platform for both optimization of injury-homing capacity and engineering for therapeutic delivery.

In the present work, we hypothesized that protein delivery by immune cells of the innate inflammatory response would allow us to target initial neuron loss during acute brain injury. We selected immature DCs as the candidate cell type for therapeutic targeting of an ischemic stroke injury because DCs are first responders to the injured brain in rodent stroke models [6], [11], [12], DCs are recruited to the human brain post-stroke [13], and given their extensive use in the immunotherapy field, DCs are readily generated ex vivo and can be modified to express foreign transgenes [14]–[16]. In the present work we find that DCs derived from a modified culture system can transiently home from the periphery to the lesioned CNS in a rat model of stroke, thus demonstrating that exogenously-derived immune cells are able to target acute CNS injury. Furthermore we find evidence that injury-responsive DCs are neuroprotective when loaded with the intracellular-acting anti-apoptotic protein Tat-BH4, and can be used to suppress neuron death during the first 24 hours after stroke, thus demonstrating the therapeutic potential of this approach.

## Results

### 1. Development of Migratory Cultured DCs

We developed a bone marrow-derived DC culture system using existing methods [14]–[16] modified to improve injury homing. We tested several in vitro and in vivo conditions for their impact on homing in the rat middle cerebral artery occlusion (MCAO) ischemia model. While existing culture methods [14]–[16] and most experimental conditions resulted in low injury-homing capacity (Figure S1, n = 120), we observed increased injury homing by DCs derived from post-natal day 11–17 bone marrow that had been cryopreserved prior to culturing (Figure S1D). Under these conditions, infusion of DCs 3 hours post-injury resulted in 4,825+/−1579 cells positive for green fluorescent protein (GFP) detectable in the ischemic hemisphere from 10 minutes to 3 hours post infusion (+/−  =  standard error of the mean, SEM, n = 24). Improved homing was associated with immature DCs, as conditions that stimulate DC maturation (e.g., treatment with a pro-inflammatory antigen, lipopolysaccharide [LPS], or transduction with endotoxin-containing lentivirus [LV]), reduced homing (Figure S1D).

**Figure 1:**
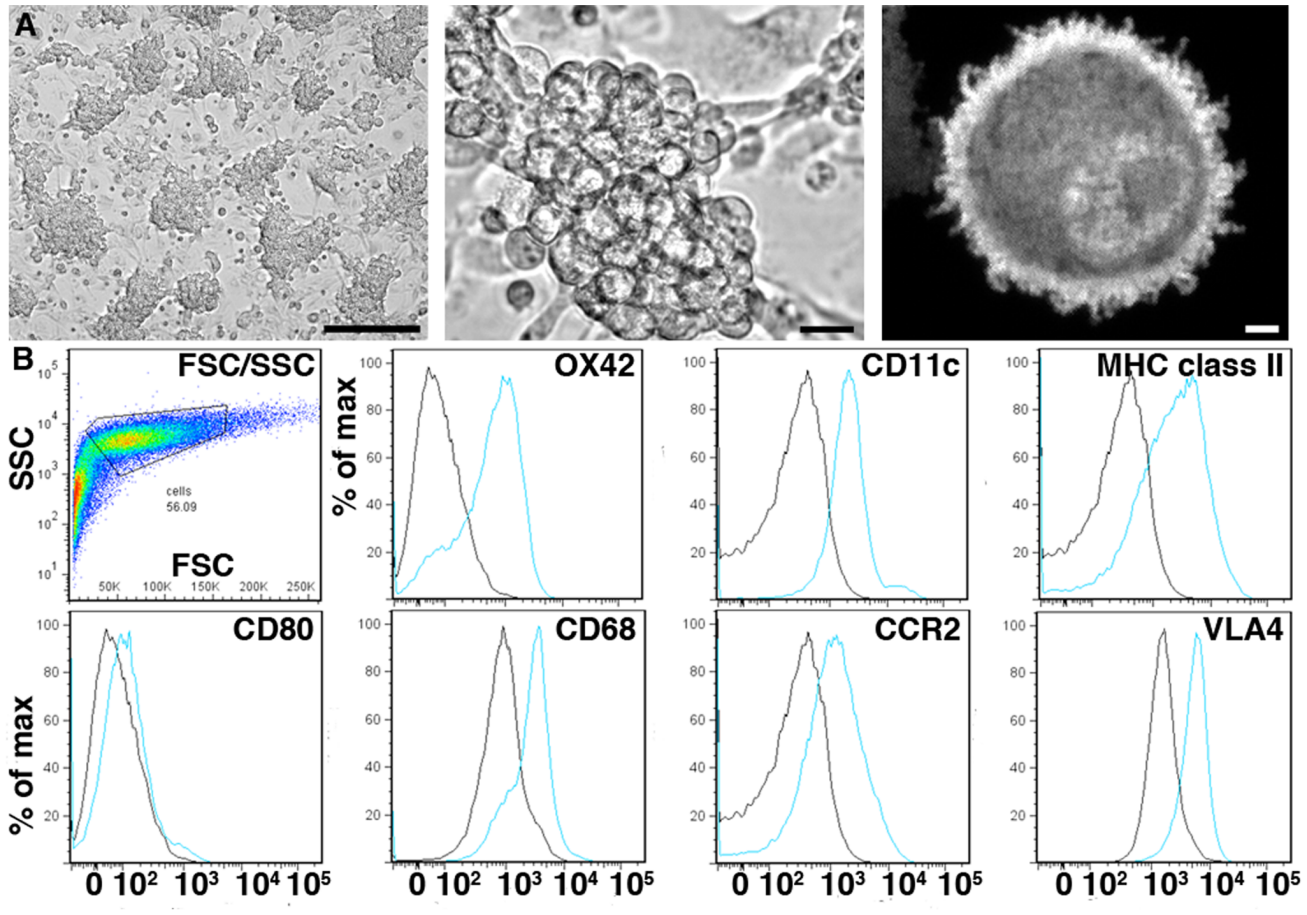
Morphological and surface marker profile of 7-day-old, lentivirus-transduced, rat bone marrow-derived DC cultures. **(A)** Large clusters of round cells predominate by culture day 7, forming a semi-adherent layer over stromal-type cells (top left and center panels). Fluorescent micrograph of a harvested, GFP-transgenic DC with representative spherical, mononuclear morphology (top right panel). Scale bars: (left) 50 µm, (center) 5 µm, (right) 0.5 µm. **(B)** Flow cytometric analysis of DC cultures showing forward- and side-scatter dot plot with the gated population prior to singlet gating (upper left panel), and histograms of mean fluorescent intensity (x-axis) versus percentage of gated population (y-axis) for CD11b/c (OX42), CD11c, MHC class II (OX6), CD80, CD68, CCR2, and VLA4. Results shown in (B) are representative of at least 3 independent experiments, assaying 20,000 cells per experiment.

Seven days of modified culture and LV transduction produced cells with spherical, mononuclear morphology consistent with immature DCs (Figure 1A) [14], [17]. Flow cytometric analysis with the myeloid lineage marker OX42 (CD11b/c), and the DC-indicative markers CD11c and MHC class II [18], indicated that 49%, 56%, and 53% of the cells expressed these markers, respectively (Figure 1B). In the absence of LPS, 3% of cells expressed the maturation marker CD80, (Figure 1B and Figure S2), consistent with the immature state of endogenous injury-responsive DCs [19]. A subset of cells expressed the lysosomal marker CD68, the inflammatory homing receptor CCR2, and the adhesion molecule very late antigen-4 (VLA-4), indicating a potential for phagocytosis, injury homing and firm binding to inflamed endothelia.

### 2. In Vivo Migration Profile of Modified DCs

**Figure 2:**
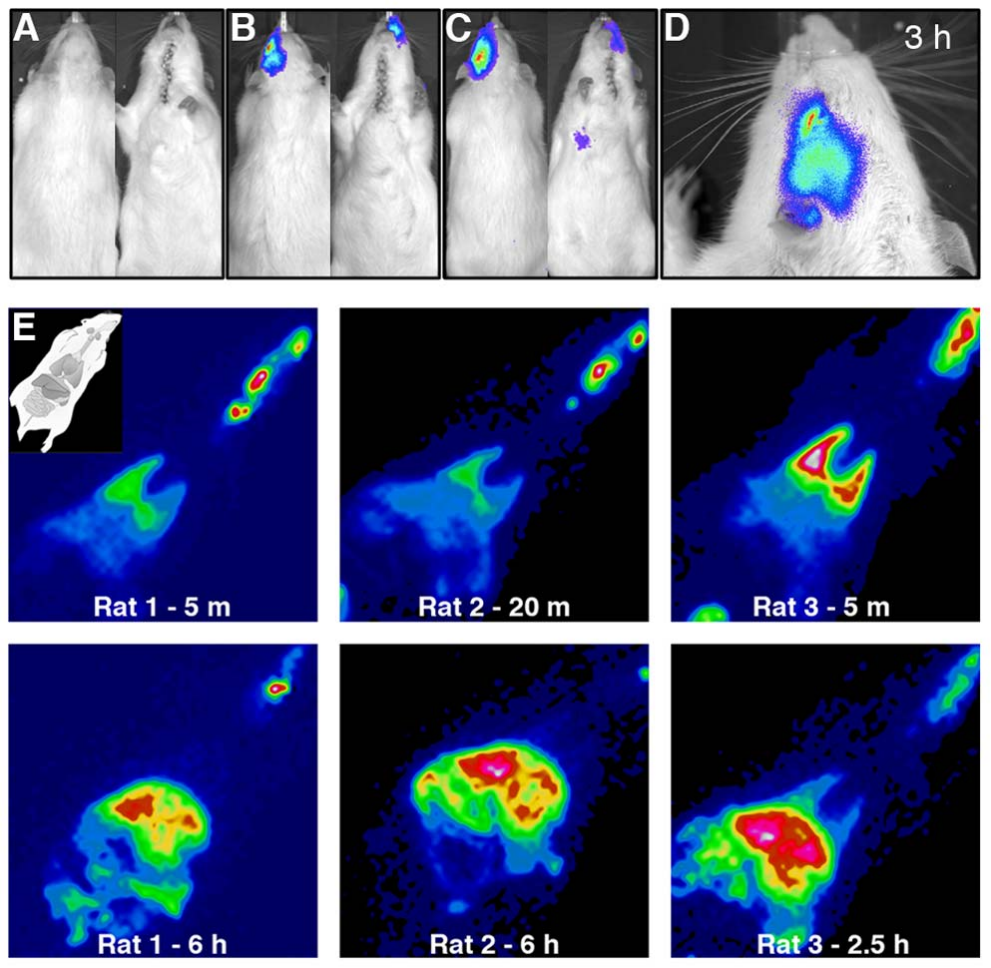
In vivo migration profile of cultured DCs. **(A)–(C)** Bioluminescent imaging of rats positioned dorsally (left panels) and ventrally (right panels) 6 h post-tMCAO and 3 h post-infusion of: **(A)** vehicle, **(B)** 2×10^6^ luciferase-DCs, or **(C)** 4×10^6^ luciferase-DCs **(D)** High resolution (low-binning) image of a luciferase-DC-infused rat at the same time point as (A)–(C). **(E)** In vivo tracking of radiolabeled DCs with SPECT. Representative images of 3 rats infused with radiolabeled DCs 3 h post-tMCAO and imaged by SPECT at 5–20 min and again at 2.5–6 h post-DC infusion. Inset in top left panel indicates the orientation of rats during imaging. Rat number (1–3) and imaging time post-DC infusion are indicated at the bottom of each image panel.

We then characterized injury-homing capacity of modified DCs. At 3 hours post-transient MCAO (tMCAO), rats received an intracarotid infusion of 2×10^6^ DCs transduced with firefly luciferase transgene, and were imaged for whole-body bioluminescence. Bioluminescence localized to the injured side of the head, persisted at 3 hours post-delivery (Figure 2A–D), and was gone by 24 hours (Figure S3). Unless the DC dose was doubled, signal was absent from the rest of the body (Figure 2C).

While this indicated that DC migration was consistent with acute homing to the injury location, limitations of bioluminescence detection in deep tissue structures prompted us to use two additional methods to track DCs in vivo. First, radiolabeling was used to characterize DC peripheral homing and to quantify their overall distribution post-infusion. At 5 minutes post-infusion, 11% of radiolabeled DCs localized to the injured side of the head, 31% to the lungs, and 26% to the liver, gut, and spleen; the remaining cells were undetectable and were presumably dispersed throughout the vasculature. At 3 and 6 hours post-infusion, 2–3% of radiolabeled DCs localized to the injured side of the head, 2–5% to the lungs, and the rest to liver, gut, and spleen (Figure 2E, n = 4).

**Figure 3:**
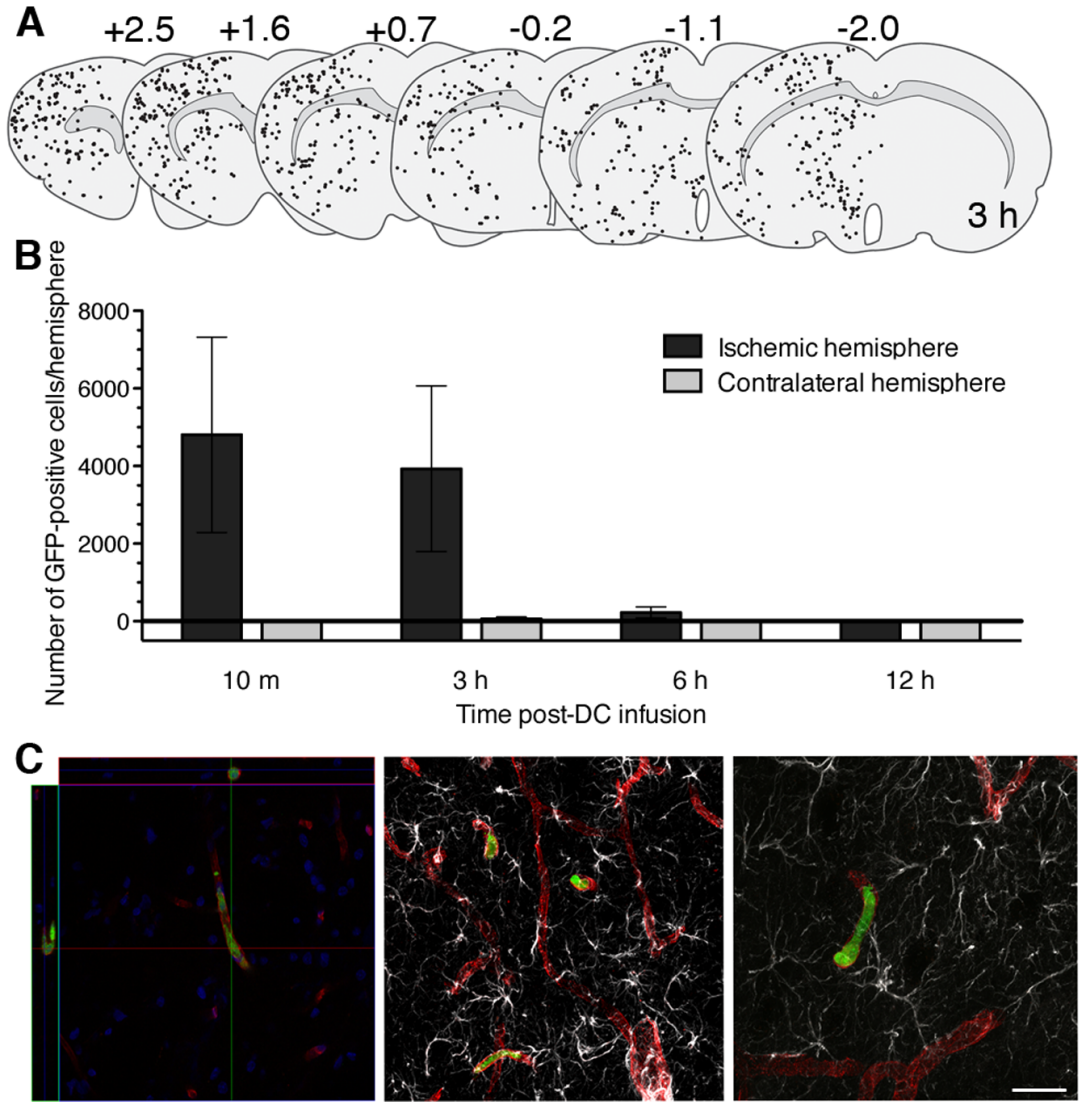
Immunofluorescent staining of GFP-positive cells at the tMCAO lesion site. **(A)** Graphical representation of GFP-positive cells in the rat brain 6 h post-tMCAO and 3 h post-DC infusion based on reconstructed whole brain section fluorescent images from a single animal. Overlying numbers indicate position relative to bregma. **(B)** Time course of GFP-positive cells in the brain following tMCAO and DC infusion. Values are expressed as the total number of GFP-positive cells per hemisphere extrapolated from counting 6 coronal sections/brain (shown in (A)). Error bars denote SEM, n = 9, 11, 5, 3 for 10 min, 3 h, 6 h, and 12 h, respectively. **(C)** Confocal images of GFP-positive cells (green) inside RECA-stained blood vessels (red). Left panel: ortho image of GFP-positive cell in the cerebrovasculature at 10 min post-cell infusion with nuclei DAPI-counterstained (blue); middle/right panel: z-stack images of GFP-positive cells in the cerebrovasculature at 3 h post cell-infusion with co-staining of GFAP-positive astrocytes (white). Scale bar: (C) 10 µm.

Next, DC homing within the ischemic hemisphere was quantified by fluorescent microscopy. GFP transgenic DCs infused 3 hours post-tMCAO were detected in the ischemic hemisphere within 10 minutes, persisted for 3 hours, and were absent by 12 hours (Figure 3A–B, S4A); few GFP-positive cells were detected contralaterally (Figure 3B). Further, immunofluorescent staining for rat endothelial cell antigen (RECA) at 10 minutes and 3 hours post-infusion showed that all GFP-positive cells were present in the injury site vasculature and were absent from brain parenchyma (Figure 3C). We then measured expression of the DC-indicative markers CD11c and MHC class II. Most recruited cells expressed CD11c and a subset expressed MHC class II (Figure S4B–C). Moreover, infused DCs accounted for most cells positive for CD11c but not MHC class II in the ischemic hemisphere 3 hours post-infusion (Figure S4D).

### 3. Development of Candidate Transgene Cargoes

**Figure 4:**
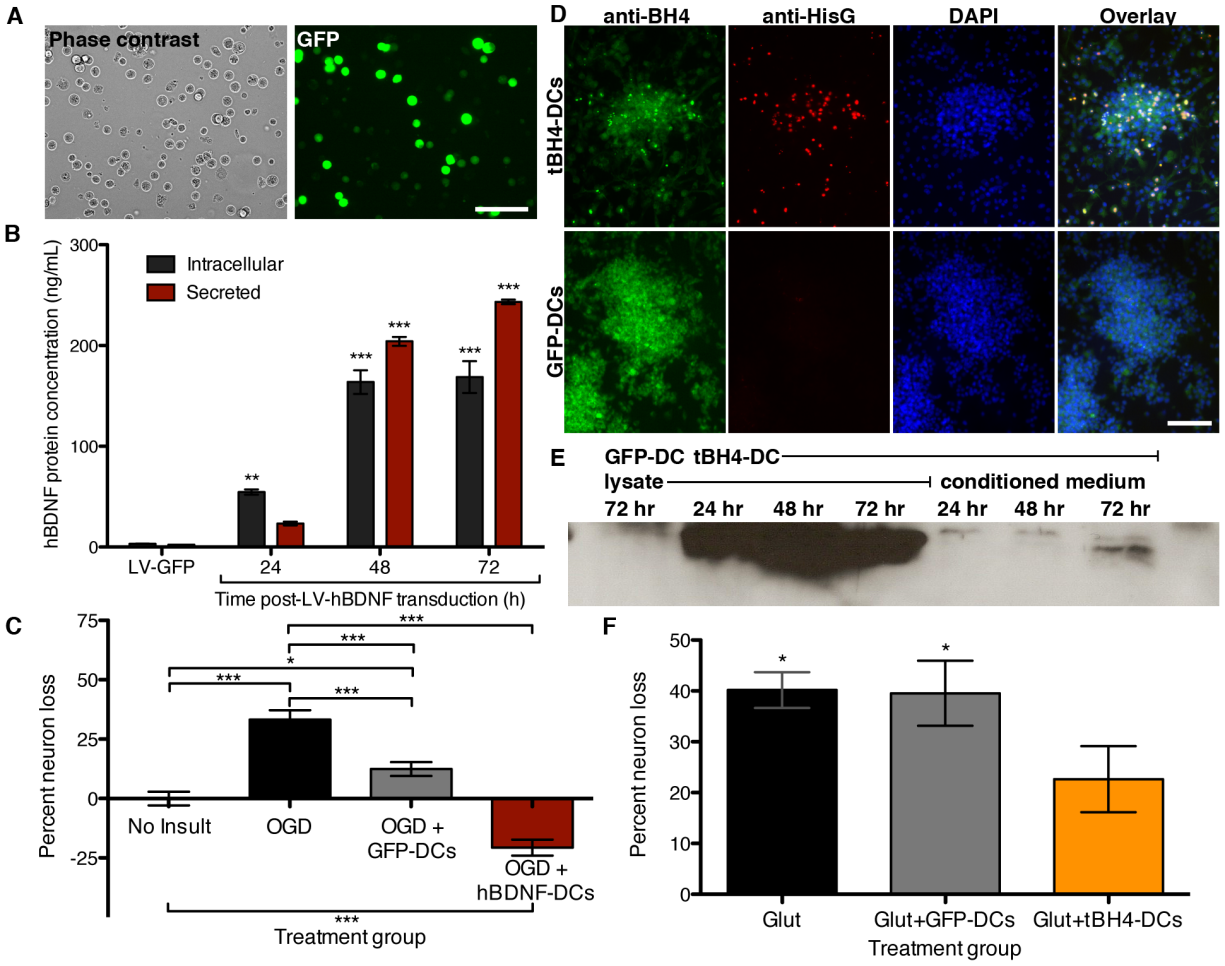
In vitro characterization of protein cargo. **(A)** Representative micrographs of harvested LV-hBDNF-transduced DCs. **(B)** Quantification of hBDNF protein in transduced-DC lysate (intracellular) and conditioned medium (secreted) by ELISA. Values reflect the combined average of 3 independent experiments, with each derived from separate lentiviral preps and DC culture preps. ** P<0.01, *** P<0.001 versus LV-GFP-transduced cultures by one-way ANOVA and Tukey post-hoc analysis. **(C)** Quantification of cortical neuron loss following OGD and DC treatment. Results were combined from at least 3 independent experiments. Error bars denote SEM. n = 46–60 wells/treatment group. * P<0.05, *** P<0.001 by one-way ANOVA and Tukey post-hoc analysis. **(D)** Representative fluorescent micrographs of LV-tBH4-transduced and LV-GFP-transduced-DC cultures. **(E)** Representative western blot showing anti-HisG immunoreactivity at 14 kDa for GFP-DC lysate or tBH4-DC lysate and conditioned medium. Time post-LV transduction is indicated for each sample. **(F)** Quantification of hippocampal neuron loss following glutamate exposure and DC treatment. Average neuron loss was calculated from at least 3 independent experiments. Error bars denote SEM. n = 24–48 wells/treatment group. * indicates significance by one-way ANOVA and Tukey post-hoc analysis with respect to each no insult control (vehicle or DC treatment alone, P<0.05). Scale bars: (A) and (D) 100 µm.

To determine if our cultured DCs could be engineered for therapeutic delivery we tested human brain-derived neurotrophic factor (hBDNF) as a candidate extracellular cargo, as post-injury delivery of hBDNF protects in stroke models [20], [21]; this effect occurs extracellularly via TrkB receptor binding [22]. DCs transduced with hBDNF (hBDNF-DCs) expressed the reporter gene GFP (Figure 4A) and hBDNF protein, as determined by enzyme-linked immunosorbent assay (ELISA) of cell lysate and conditioned medium (Figure 4B).

We then tested the therapeutic efficacy of hBDNF-DCs with rat primary cortical neuron cultures undergoing oxygen-glucose deprivation (OGD). OGD treatment for 6 hours followed by 18 hours of reperfusion caused 30% loss of the neuronal marker MAP2 [23] (Figure 4C). Pilot studies indicated that up to 1000 transduced DCs could be added to neuron cultures without any significant neurotoxicity (Figure S5), and when added at the onset of reperfusion, hBDNF-DCs blocked OGD neurotoxicity and even enhanced neuron survival relative to uninjured control cultures (Figure 4C). Unexpectedly, control GFP-DCs also reduced OGD-induced neuron loss, but to a lesser extent than hBDNF-DCs (Figure 4C).

While neurotrophins and other extracellular proteins can be protective, numerous steps in the neuron death cascade can be blocked by factors acting intracellularly [24]. Thus, we developed a second intracellular-acting protein cargo that was modified for transport across both the BBB and cell membranes. To allow for transcellular transport, the protein cargo was designed to contain the HIV-derived trans-activator of transcription (Tat) domain [25], [26]. We designed a secretable protein consisting of Tat and the BH4 domain of the intracellular anti-apoptotic protein Bcl-xL (Tat-BH4); prior work demonstrated neuroprotection by Tat-BH4 or Tat-Bcl-xL both in vitro and in vivo [27]–[29]. DC cultures transduced to express Tat-BH4 (tBH4-DCs) exhibited anti-BH4 staining co-localized with a HisG tag present in the Tat-BH4 fusion protein, whereas, DCs transduced with a control vector were BH4-positive only, presumably reflecting endogenous Bcl-xL protein (Figure 4D). Further, HisG reactivity was detectable by western blot in lysate and conditioned medium of tBH4-DCs; this band was absent from control samples (Figure 4E). We then tested the therapeutic efficacy of tBH4-DCs with primary hippocampal neuron cultures subject to glutamate excitotoxicity, as purified Tat-BH4 protein protects in this paradigm [28]. There was 40% neuron death with 50 µM glutamate, and simultaneous treatment with tBH4-DCs, but not GFP-DCs, lessened the toxicity (Figure 4F).

Because DCs are known to have potent immunoregulatory function, we measured the ability of transgene-loaded DCs to stimulate T cell proliferation in a mixed leukocyte reaction (MLR). Interestingly, DCs modified for injury homing exhibited reduced T cell activation relative to DCs derived from adult rat bone marrow and standard culture conditions, and this reduction was greatest for tBH4-DCs (Figure S6). For both culture methods, T cell activation increased when DCs were pre-treated with LPS (1 µg/mL), but the total amount of T cell activation remained significantly lower for transgene-loaded, modified DCs (Figure S6).

### 4. In Vivo Efficacy of Cargo-Loaded DCs

**Figure 5:**
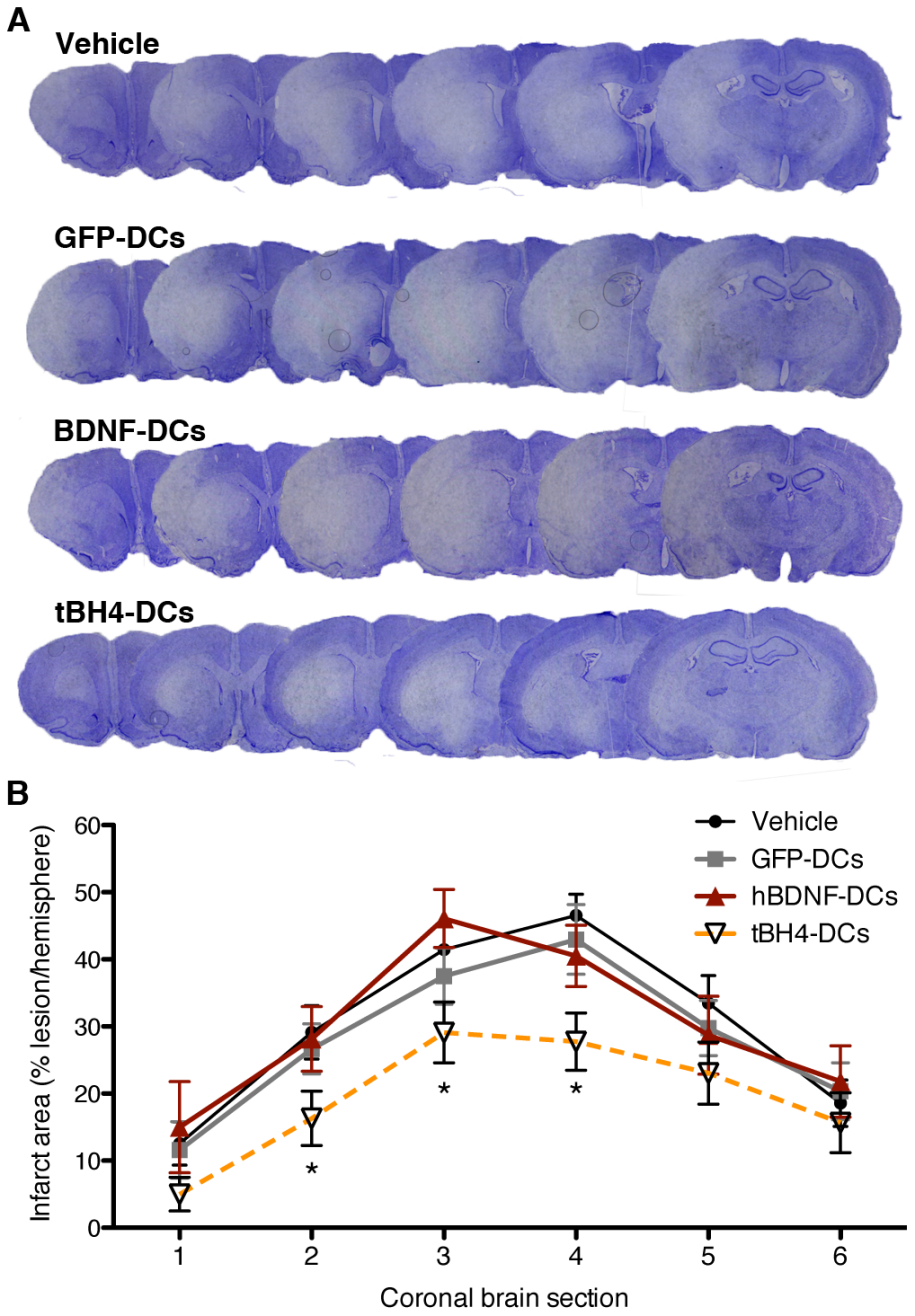
Infarct quantification following treatment with protein-loaded DCs. **(A)** Representative cresyl violet stain for each treatment group showing the 6 coronal sections used to quantify lesion size at 24 h post-tMCAO. **(B)** Quantification of lesion size at 24 h post-tMCAO by coronal brain section and treatment group. * indicates significance by one-way ANOVA plus Student-Newman-Keuls post-hoc analysis of tBH4-DCs and GFP-DCs for the sum infarct area of coronal sections 2–4, P = 0.040.

We then tested the ability of our system to prevent tMCAO-induced neuron death. At 3 hours post-tMCAO, rats were infused with vehicle, GFP-DCs, hBDNF-DCs, or tBH4-DCs and infarct was measured by Nissl stain 24 hours post-tMCAO. For vehicle- or GFP-DCs-infused rats, there was near complete loss of the striatum and partial loss of the cortex (Figure 5A). In contrast to its protective effect in vitro, infusion of hBDNF-DCs was not protective (Figure 5B, S7A). However, infusion of tBH4-DCs decreased infarct area by ∼33% (Figure S7A) along the anterior-posterior axis of the lesion (Figure 5A–B). In the cortex, or penumbral region of the infarct, tBH4s-DCs reduced damage by ∼50% (Figure S7B–C). Neither GFP-DCs nor tBH4-DCs altered the number of phagocytic immune cells at the lesion site at 24 hours (number of cells in the ischemic hemisphere positive for the pan-immune cell marker CD68: vehicle = 541.9+/−83.5, GFP-DC = 427.1+/−72.9, tBH4-DC = 497.1+/−71.4, n = 8).

## Discussion

Therapies targeting neurological disease are limited by the need for improved means of delivery. Gene therapy typically requires invasive neurosurgery and direct viral transduction of target cells or long-term implantation of gene-modified carrier cells. Alternatively, therapies involving systemic delivery of recombinant proteins, small molecules, naked DNA or liposomes containing DNA to the injury site are less invasive, but lack specificity in and to the brain. As way to address this, we developed a non-invasive protein delivery system using cultured, transgene-loaded DCs.

We selected DCs as they are among the first-responders to neurological injury [6], [11], [12] and thus could intervene rapidly post-injury. We developed culturing conditions to produce immature, injury-responsive DCs, and when infused intra-arterially 3 hours post-stroke, these cells transiently homed to the injury site, arriving within 10 minutes and persisting in the surrounding microvasculature for up to 6 hours (Figure 2, Figure 3). This timing coincides with upregulation of the chemokines monocyte chemoattractant protein-1 (MCP-1) and cytokine-induced neutrophil chemoattractant-1 (CINC-1) [30] and endothelial adhesion molecules P- and E-selectin in the ischemic hemisphere [31], as well as injury targeting by endogenous DCs [11]. Based on radiolabeling studies, modified DC culture conditions resulted in approximately 11% of the infused population homing to the injury site immediately after intracarotid delivery, and this decreased to 2–3% by 3 hours post-delivery (Figure 2E). Thus, additional work is needed to optimize cultured DCs as efficient drug vectors, and this might be achieved by selective enrichment of the injury-homing population, either by further modification of the cell culture conditions, and/or cell sorting. Indeed, preliminary mass spectrometry analysis of modified DCs showed a membrane-associated protein profile distinct from standard DC cultures, including candidate surface proteins for negative sorting, such as MHC class II and Vinculin (Dataset S1).

The potent immunoregulatory capacity of DCs [17], [18] is a major consideration with this approach. DC infusion 3 hours post-tMCAO did not affect infarct size or recruitment of CD68-positive immune cells at 24 hours (Figure 5), suggesting that DC treatment did not significantly alter the initial innate immune response to stroke injury within this time frame. The preferential localization of DCs to the injury site vasculature (Figure 3C) may partially explain this, as CNS infiltration by immune cells can worsen inflammation and injury [32], [33]. In addition to their capacity for transient injury homing, modified DCs exhibited characteristic homing to peripheral organs, including spleen, during the first 6 hours post-infusion (Figure 2E) [34], therefore raising significant concerns about a potential T cell response following DC treatment. We tested the capacity of modified DCs to stimulate T cell proliferation in vitro by MLR, and found that relative to DCs derived from standard culture conditions, modified DCs had reduced T cell stimulatory capacity, which was further decreased by DC expression of the fusion transgene Tat-BH4 (Figure S6). Nonetheless, whether this approach can influence adaptive immunity in vivo is a crucial focus for future studies in order to identify any potential host responses to DC treatment and/or a given transgene cargo. To circumvent this, future studies may explore use of syngeneic DCs, co-administration of cargo-loaded tolerogenic DCs, or modification of DCs to include either an inducible suicide switch (e.g., the gene for cytosine deaminase [35]) to clear DCs or a means to suppress DC antigen presentation such as RNA interference.

Traditional CNS delivery strategies have shown efficacy with both intracellular- and extracellular-acting factors [24], [36]. We tested DC-based delivery with two candidate cargos: hBDNF and the intracellular anti-apoptotic factor BH4. DCs loaded with hBDNF protected against OGD in vitro (Figure 4C), but not against stroke in vivo (Figure 5). This may reflect a need for sustained delivery and transport of BDNF into the CNS parenchyma, as either stem cell-mediated delivery [21], [37], or systemic delivery of BDNF conjugated to a BBB transferrin receptor antibody [20] reduced stroke injury. In contrast, DC delivery of the anti-apoptotic protein BH4 fused with membrane-permeable Tat protected against excitotoxicity in vitro and reduced ischemic brain damage 24 hours after tMCAO in vivo (Figure 4F, Figure 5). In vivo protection was greatest in the cortex, corresponding to the penumbral zone of injury where a significant proportion of cells die apoptotically (Figure 5A, Figure S7B–C) [38]. This extent of infarct reduction with tBH4-loaded, modified DCs was similar to that of systemically-delivered stem cells or therapeutic proteins [39], [40].

While it is possible that this effect resulted from Tat-mediated delivery of BH4 into target cells at the injury site [25], [41], neuroprotection may also have resulted indirectly from tBH4-DC activity in peripheral organs, as might be expected if, for example, tBH4-DC treatment caused a reduced inflammatory response in spleen [42], [43]. In this case, future studies might explore the use of this system for therapeutic delivery to both the injured brain and peripheral organs. Moreover, differential targeting of DCs to the injured brain and periphery might be achieved through selective isolation of migratory DC sub-populations. Whether this approach can impact stroke injury progression and recovery beyond the first 24 hours will be an important focus for future studies, particularly with respect to its potential use for delivery of recovery enhancing therapeutic cargoes.

## Materials and Methods

### Ethics Statement

All procedures used in this study were approved by the Stanford University Administrative Panel on Laboratory Care (Protocol # 10873, 10443) and the Association for Assessment of Laboratory Animal Care and are in compliance with the National Institute of Health Guide for the Care and Use of Laboratory Animals.

### Bone marrow-derived DC cultures

DC cultures were generated as described previously [14]–[16] from SD or GFP-transgenic rat pup bone marrow (post-natal day 11–17). Following liquid nitrogen storage, cells were seeded in 6-well plates at 2×10^6^ cells/mL in complete medium (CM) containing the cytokines, interleukin-4 (IL-4), granulocyte-macrophage colony stimulating factor (GM-CSF), and Flt-3 ligand (5 ng/mL/cytokine, R&D Systems). On DIV 4, medium containing non-adherent cells was removed and replaced with CM containing protamine sulfate (10 µg/mL, Sigma), and vehicle or LV (multiplicity of infection = 10–15). After 18 hours, medium was replaced with CM (75% new/25% conditioned). On DIV 6–7, cultures were harvested with PBS.

### LV vector construction and purification

LV particles were generated as described previously [44]. Transfer vectors containing GFP, with or without a multiple cloning site (MCS) and internal ribosomal entry site (IRES) preceding GFP (pHR-IG and pHR-G, respectively), along with production plasmids were a gift from C. Garrison Fathman. The following transgenes were inserted at the MCS of pHR-IG: firefly luciferase (Genbank: X84848); human BDNF (isoform A, Genbank: NM_001143816; gift from R. Tolwani); Tat-BH4 containing rat fibronectin signal secretory sequence (nt.: 210–305, Genbank: NM_019143.2), 6xhistidine tag, Tat domain from HIV gag (a.a.: 48–58, Genbank: HM027826.1), BH4 domain of rat Bcl-xL (a.a. 4–23, Genbank: AF136230), flag tag, stop codon.

### Flow cytometry

DIV 7 cells were harvested (permeabilized for CD68), blocked with 50% FBS in FACS buffer (FB, 1mM EDTA, 1% FBS in PBS) and incubated with primary antibodies (Ox42-biotin (Serotec); CD11c (AbCam); MHC classII (Serotec); CD80-biotin (eBioscience); hCCR2 (R&D System); VLA4-Alexa647 (Serotec); CD68-Alexa647 (AbD Serotec); mouse IgG1 negative control-Alexa647 (Serotec)). For unconjugated antibodies, cells were then incubated with secondary antibodies (streptavidin-PECy7 (Invitrogen); goat-anti-mouse-IgG-RPE (Serotec)). Cells were fixed in 1% paraformaldehyde (PFA), run on an LSR II flow cytometer (BD) at the Stanford Shared FACS Facility and analyzed using FlowJo (Tree Star, Inc.).

### In vitro detection of hBDNF and Tat-BH4

DCs transduced with LV-hBDNF, LV-tBH4, LV-GFP, or vehicle (CM + protamine sulfate) and conditioned medium were collected at 24, 48, or 72 hours post-transduction. Total cellular protein was extracted [45] and quantified using the BCA Protein Assay (Pierce). hBDNF protein was quantified with the DuoSet hBDNF ELISA kit (R&D Systems) and normalized to total protein. For Tat-BH4 detection, LV-GFP- or LV-tBH4-transduced cultures (DIV 7) were fixed with cold methanol and stained with rabbit-anti-BH4 (Santa Cruz), mouse-anti-HisG (Invitrogen), and AlexaFluor-conjugated secondary antibodies (Invitrogen). Tat-BH4 protein in DC conditioned medium and cell lysate was detected by western blot (anti-HisG, Invitrogen) as described previously [45]. For each assay, protein was collected from 3 separate culture preps independently transduced with the appropriate LV vector.

### Primary neuronal cultures

Mixed neuronal/glial cortical or hippocampal cultures were prepared from day 18 fetal SD rats [46], [28]. On DIV 12, cortical cultures were treated with OGD and hippocampal cultures were treated with glutamate [46], [28]. Following OGD or concomitant to glutamate exposure, neuronal cultures were treated with vehicle (MEM, Gibco), GFP-DCs, hBDNF-DCs, or tBH4-DCs (1000 DCs/well) and assayed for neuron death 24 hours post-insult onset [47]. For both assays, DCs were incubated in vehicle at a density of 1000 DCs/ µL for 24 hours prior to neuron co-culture.

### tMCAO stroke model and intravascular DC delivery

Male SD rats (300–340 g) were subject to 1 hour tMCAO as described previously [48], using a silicon-tipped 4-0 monofilament (Doccol). Three hours post-reperfusion, vehicle (50% RPMI-1640/50% CM (without cytokines) or 2×10^6^ DCs were infused via a catheter (PE10, BD) into the ECA-ICA junction as a 0.3 mL infusion at 0.1 mL/min.

### Tissue processing

Animals were perfused transcardially with 0.9% saline and 4% PFA. Brains were cryoprotected in 20% sucrose/4% PFA for 24 hours, stored at −80°C. For animals subject to tMCAO, sets of 6 coronal sections spanning the infarct region were collected (anterior/posterior to bregma: +2.5 to −2.0 mm, 0.9 mm increments between each section). At 3 hours post-cell infusion, co-localization of GFP fluorescence was quantified with anti-GFP (Invitrogen), anti-ratCD11c (AbCam), and anti-ratOX6 (Serotec) by 2 blinded observers (n = 4 animals/group, approximately 50 cells counted/animal). At 24 hours post-reperfusion, CD68 (BD) staining in the infarcted hemisphere was counted by a blinded observer (n = 8/group). At 10 minutes or 3 hours post-cell infusion, free-floating sections were stained with RECA (AbCam) and GFAP (AbCam).

### Infarct quantification

Lesion size was quantified 24 hours post-reperfusion by Nissl stain. A blinded observer analyzed infarct area with ImageJ Software (NIH) comparing total infarct area across 6 representative coronal sections for vehicle-, GFP-DC, tBH4-DC, and hBDNF-DC-infused animals The infarct sum of coronal sections 2–4 for vehicle-, LV-GFP-, and LV-tatBH4-injected animals was further compared; this region exhibited the most consistent damage across animals. n = 14, 15, 15, 17 for vehicle, GFP-DCs, hBDNF-DCs, and tBH4-DCs, respectively.

### Statistics

Results were analyzed using Prizm 5.0 (GraphPad) as follows, hBDNF ELISA, neuron loss with OGD or glutamate, and MLR: one-way ANOVA and Tukey post-hoc analysis; infarct quantification: two-tailed t-tests; infarct quantification of coronal sections 2–4:one-way ANOVA and Student-Newman-Keuls post-hoc analysis; CD68 quantification: one-way ANOVA.

Refer to Text S1 for detailed methodology and Figure S8 for a map of the lentiviral vector constructs.

## Supporting Information

Figure S1 **Identification of in vitro and in vivo parameters found to influence the injury-homing capacity of cultured DCs.** **(A)** Schematic indicating the brain regions shown in (B) and (C). **(B)** Representative fluorescent micrograph showing low injury-homing capacity to the tMCAO-lesioned cortex at 6 h post-injury, 3 h post-cell infusion. **(C)** Representative fluorescent micrograph showing enhanced injury-homing capacity to the tMCAO-lesioned cortex at 6 h post-injury, 3 h post-cell infusion. **(D)** Table of parameters found to influence injury-homing capacity: conditions shown in the center column were associated with low injury-homing capacity (<10 GFP-positive cells in 6 representative sections spanning the lesion site in 94% of animals, n = 3–6 animals/condition, total n = 120), whereas combined use of conditions shown in the right column was sufficient to increase this homing (4,825+/−1579 GFP-positive cells extrapolated from quantification of 6 representative sections spanning the lesion site, SEM, n = 25). Abbreviations: GM-CSF  =  granulocyte-macrophage colony stimulating factor; IL-4  =  interleukin-4; Flt-3  =  fms-like tyrosine kinase 3; EDTA  =  ethylenediametetraacetic acid; *tMCAO  =  transient middle cerebral artery occlusion via external carotid artery filament insertion; tMCAO  =  transient MCAO via common carotid artery filament insertion; dMCAO  =  distal MCAO via electrocoagulation of the MCA; ICA  =  internal carotid artery. Scale bar in (C) 100 µm.
(TIF)

Figure S2 **Flow cytometric analysis of bone marrow-derived DC cultures treated with LPS.** Representative histograms showing mean fluorescent intensity (x-axis) vs. percentage of gated cells (y-axis) for OX42, CD11c, MHC class II (OX6), CD80, CCR2, and VLA-4 for 7-day-old DC cultures. Black lines correspond to fluorescent signal from isotype controls. Blue lines correspond to control DC cultures. Red lines correspond to DC cultures treated with 0.3 µg/mL LPS on DIV 6.
(TIF)

Figure S3 **In vivo bioluminescent tracking of luciferase-transduced DCs.** Representative images of 2 rats infused with luciferase-DCs 3 h post-tMCAO and imaged at 3 h and again at 24 h post-DC infusion. For each rat/imaging time point, adjacent dorsal and ventral views are displayed. Rat number and imaging time post-DC infusion is indicated in the top of each pair of dorsal/ventral images.
(TIF)

Figure S4 **Immunofluorescent staining of GFP-positive cells at the tMCAO lesion site.** **(A)** Confocal fluorescent micrographs of anti-GFP staining in the ischemic hemisphere 6 h post-tMCAO and 3 h post-DC infusion. **(B)** GFP-positive cells at the lesion site 3 h post-cell infusion co-labeled and overlayed with CD11c or MHC class II (OX6). Nuclei are counterstained with DAPI in the overlay panels. **(C)** Quantification of GFP-positive cells co-labeled with CD11c and MHC class II. Error bars denote SEM. n = 4 animals per surface marker with an average of 128 cells/animal scored for CD11c and an average of 27 cells/animal scored for MHC class II. (D) Quantification of total CD11c and MHC class II expression in the ischemic hemisphere of rats 6 h post-tMCAO and 3 h post-DC infusion. The relative contributions of endogenous cells versus infused DCs on the expression of each surface marker was determined by the percentage of GFP-negative/surface marker-positive cells versus the percentage of cells double-positive for GFP and each surface marker. n = 4 animals/surface marker with an average of 50 cells counted per surface marker per animal. Error bars denote SEM. Scale bars: (A) 50 µm.
(TIF)

Figure S5 **Relative neurotoxicity of transduced DCs in vitro.** Quantification of rat primary cortical neuron survival 24 h after exposure to increasing doses of **(A)** DCs transduced with LV-GFP or **(B)** DCs transduced with LV-hBDNF. *** P<0.001; * P<0.05 by one-way ANOVA and Tukey post-hoc analysis. n = 48 wells for 0 and 1000; n = 6 wells for 500; n = 18 wells for 5000; n = 12 wells for 10,000. Previously, we found that our rat primary neuron cultures grown in this format contain approximately 10,000–20,000 neurons/well [47] suggesting that in vitro ratios of approximately 1∶40 to 1∶10 transduced DCs:neurons were not neurotoxic, whereas higher ratios of DCs:neurons were neurotoxic.
(TIF)

Figure S6 **Relative in vitro T cell stimulatory capacity of optimized, transgene loaded-DCs.** DCs derived from adult rat bone marrow using standard methods (adult) or from pup bone marrow using the modified method (pup) were tested for their ability to stimulate T cell proliferation in a mixed leukocyte reaction (MLR). LPS-treated DCs were used as a positive control and T cells alone or mitomycin C-treated DCs were used as negative controls. Values represent combined average of wells with DC:T cell ratios of 1∶16 and 1∶8 (6 wells/value). In addition to DC culture type, DC pretreatment with LPS and/or transgene cargo loading is indicated below the x-axis. ***/*** indicates significance by one-way ANOVA plus Tukey post-hoc analysis relative to adult DCs (black asterisks) or relative to adult DCs+LPS (green asterisks). n = 6 wells/group. Error bars denote SEM.
(TIF)

Figure S7 **Quantification of ischemic damage following treatment with protein-loaded DCs.** **(A)** Quantification of infarct area for each treatment group, expressed as the percent damage/total hemisphere area. * indicates significance by two-tailed t-test, P = 0.034. Error bars denote SEM. n = 14, 15, 15, 17 for vehicle, GFP-DCs, hBDNF-DCs, and tBH4-DCs, respectively. **(B)** Quantification of cortical damage for each treatment group at 24 h post-tMCAO, expressed as the percent damage/total cortical area. * indicates significance by two-tailed t-test, P = 0.019. Error bars denote SEM. n = 14, 15, 15, 17 for vehicle, GFP-DCs, hBDNF-DCs, and tBH4-DCs, respectively. **(C)** Quantification of percent cortical damage at 24 h post-tMCAO by coronal brain section and treatment group. * indicates significance by one-way ANOVA plus Student-Newman-Keuls post-hoc analysis of tBH4-DCs and vehicle treatment for the sum of cortical damage for coronal sections 2–4, P = 0.017. Using the same statistical analysis of cortical damage in coronal sections 2–4 indicated a strong trend towards protection for tBH4-DCs compared to GFP-DC (P = 0.051). Error bars denote SEM.
(TIF)

Figure S8 **Plasmid map for lentiviral backbone, pHR-IG, and transgene inserts.** Plasmid features are indicated with arrows. Abbreviations: CMV  =  cytomegalovirus; MCS  =  multiple cloning site; IRES  =  internal ribosomal entry site; eGFP  =  enhanced green fluorescent protein; LTR  =  long terminal repeat; amp R  =  ampicillin resistance gene; pUC  =  origin of replication; SV40 prom-enh  =  simian virus 40 promoter-enhancer; gpt  =  guanine-hypoxanthine phosphoribosyl transferase; psi  =  viral packaging sequence; x = 5′ and 3′ splice sites; RRE  =  rev-responsive element; sss  =  signal secretory sequence.
(TIF)

Text S1 **Supporting Materials and Methods.**
(DOC)

Dataset S1 **Mass spectrometry analysis of standard and modified DC cultures.**
(XLS)
