# Supplementary figures and images for: Derivation of Injury-Responsive Dendritic Cells for Acute Brain Targeting and Therapeutic Protein Delivery in the Stroke-Injured Rat
Source: PLoS One. 2013 Apr 16;8(4):e61789. doi: 10.1371/journal.pone.0061789 (PMC3627911; doi:10.1371/journal.pone.0061789)

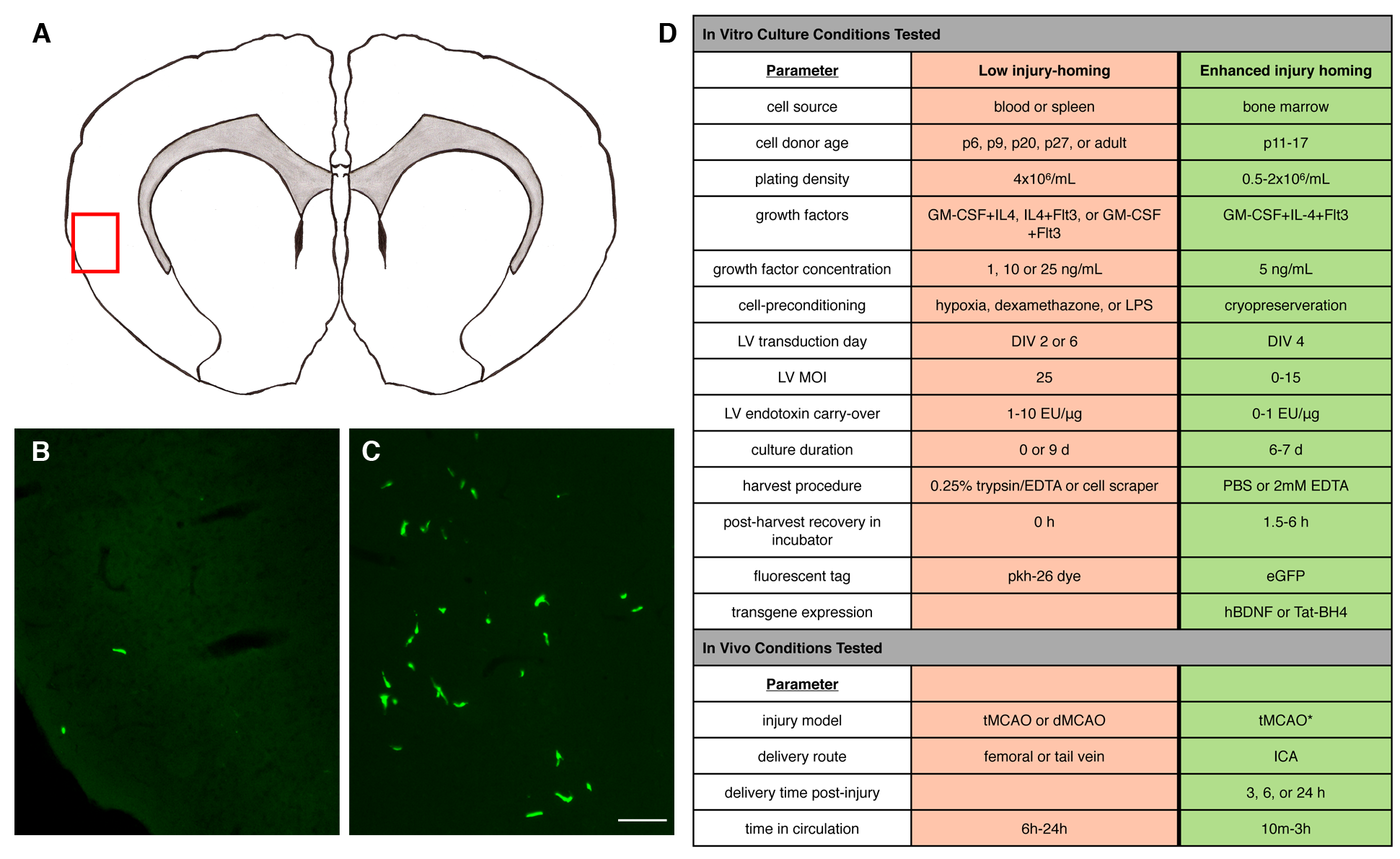

Supplement: Figure S1 — Identification of in vitro and in vivo parameters found to influence the injury-homing capacity of cultured DCs. (A) Schematic indicating the brain regions shown in (B) and (C). (B) Representative fluorescent micrograph showing low injury-homing capacity to the tMCAO-lesioned cortex at 6 h post-injury, 3 h post-cell infusion. (C) Representative fluorescent micrograph showing enhanced injury-homing capacity to the tMCAO-lesioned cortex at 6 h post-injury, 3 h post-cell infusion. (D) Table of parameters found to influence injury-homing capacity: conditions shown in the center column were associated with low injury-homing capacity (<10 GFP-positive cells in 6 representative sections spanning the lesion site in 94% of animals, n = 3–6 animals/condition, total n = 120), whereas combined use of conditions shown in the right column was sufficient to increase this homing (4,825+/−1579 GFP-positive cells extrapolated from quantification of 6 representative sections spanning the lesion site, SEM, n = 25). Abbreviations: GM-CSF = granulocyte-macrophage colony stimulating factor; IL-4 = interleukin-4; Flt-3 = fms-like tyrosine kinase 3; EDTA = ethylenediametetraacetic acid; *tMCAO = transient middle cerebral artery occlusion via external carotid artery filament insertion; tMCAO = transient MCAO via common carotid artery filament insertion; dMCAO = distal MCAO via electrocoagulation of the MCA; ICA = internal carotid artery. Scale bar in (C) 100 µm. (TIF) [file pone.0061789.s001.tif]

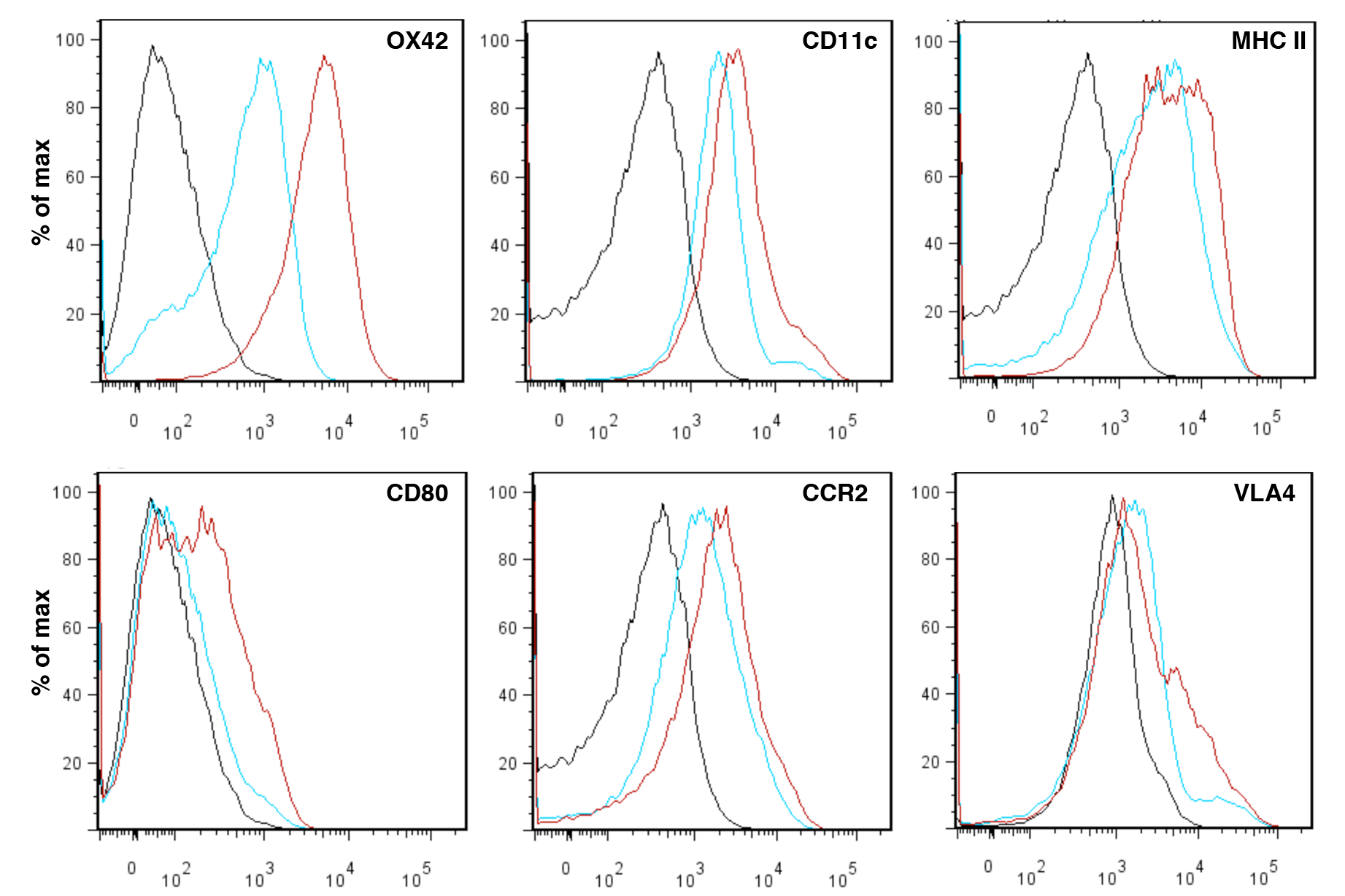

Supplement: Figure S2 — Flow cytometric analysis of bone marrow-derived DC cultures treated with LPS. Representative histograms showing mean fluorescent intensity (x-axis) vs. percentage of gated cells (y-axis) for OX42, CD11c, MHC class II (OX6), CD80, CCR2, and VLA-4 for 7-day-old DC cultures. Black lines correspond to fluorescent signal from isotype controls. Blue lines correspond to control DC cultures. Red lines correspond to DC cultures treated with 0.3 µg/mL LPS on DIV 6. (TIF) [file pone.0061789.s002.tif]

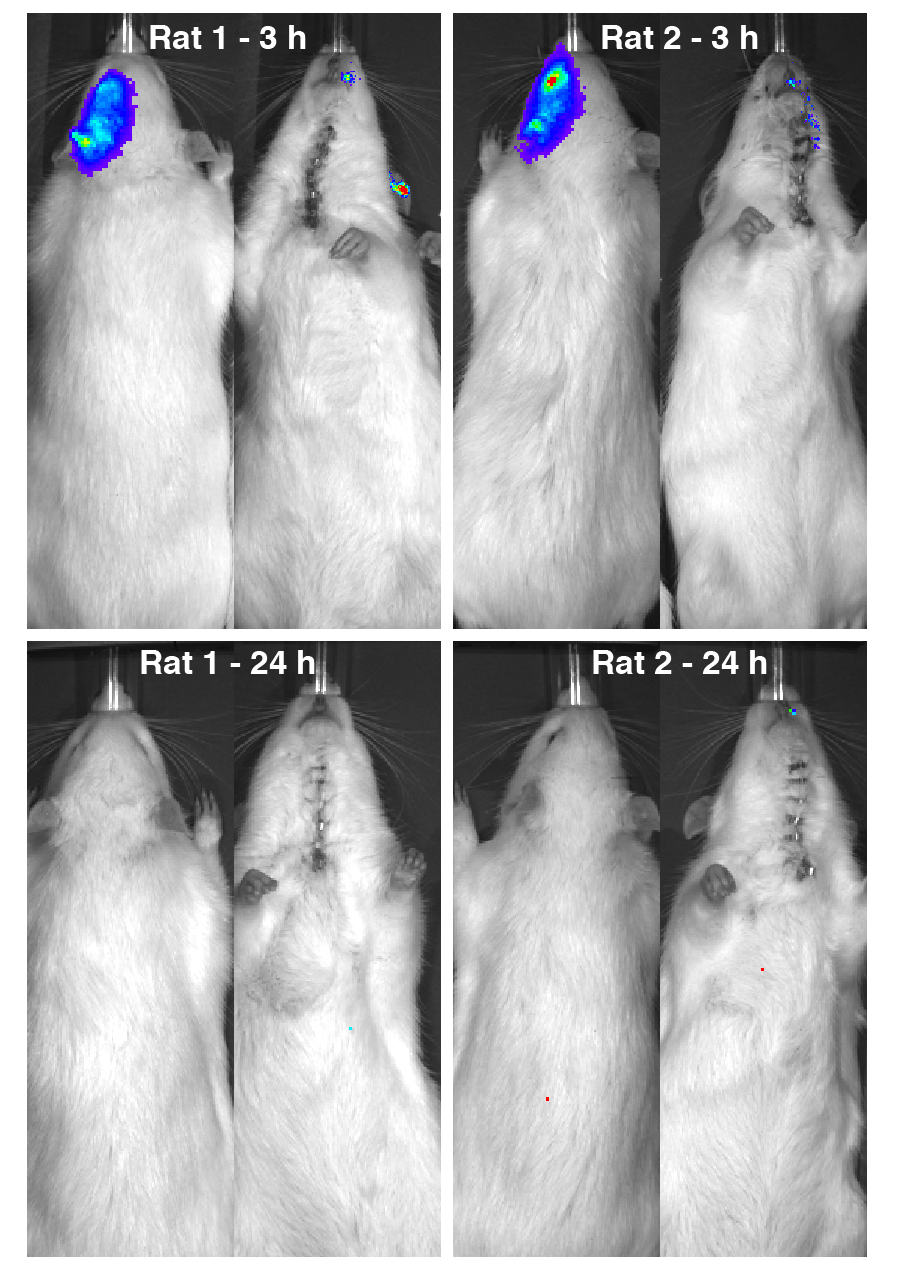

Supplement: Figure S3 — In vivo bioluminescent tracking of luciferase-transduced DCs. Representative images of 2 rats infused with luciferase-DCs 3 h post-tMCAO and imaged at 3 h and again at 24 h post-DC infusion. For each rat/imaging time point, adjacent dorsal and ventral views are displayed. Rat number and imaging time post-DC infusion is indicated in the top of each pair of dorsal/ventral images. (TIF) [file pone.0061789.s003.tif]

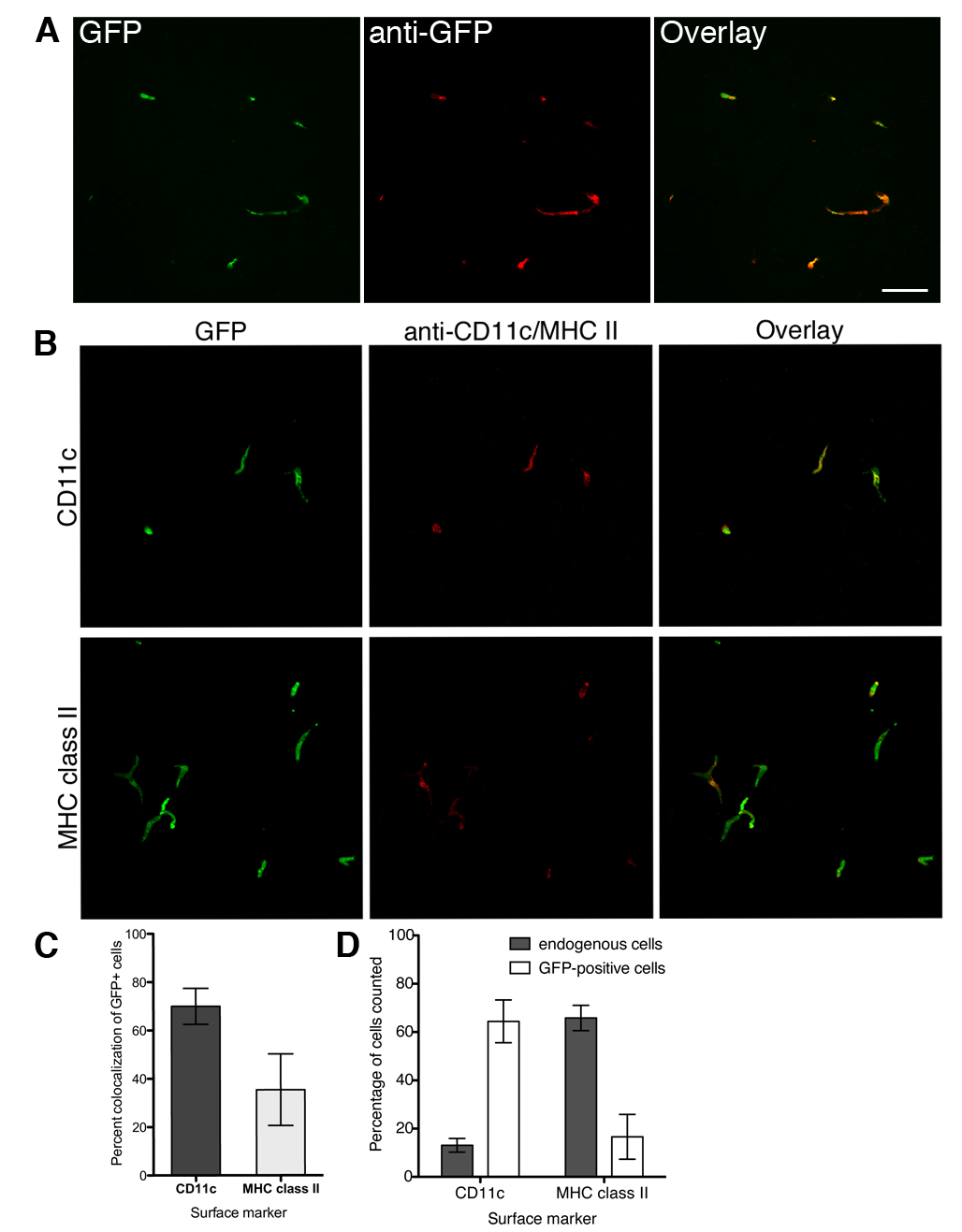

Supplement: Figure S4 — Immunofluorescent staining of GFP-positive cells at the tMCAO lesion site. (A) Confocal fluorescent micrographs of anti-GFP staining in the ischemic hemisphere 6 h post-tMCAO and 3 h post-DC infusion. (B) GFP-positive cells at the lesion site 3 h post-cell infusion co-labeled and overlayed with CD11c or MHC class II (OX6). Nuclei are counterstained with DAPI in the overlay panels. (C) Quantification of GFP-positive cells co-labeled with CD11c and MHC class II. Error bars denote SEM. n = 4 animals per surface marker with an average of 128 cells/animal scored for CD11c and an average of 27 cells/animal scored for MHC class II. (D) Quantification of total CD11c and MHC class II expression in the ischemic hemisphere of rats 6 h post-tMCAO and 3 h post-DC infusion. The relative contributions of endogenous cells versus infused DCs on the expression of each surface marker was determined by the percentage of GFP-negative/surface marker-positive cells versus the percentage of cells double-positive for GFP and each surface marker. n = 4 animals/surface marker with an average of 50 cells counted per surface marker per animal. Error bars denote SEM. Scale bars: (A) 50 µm. (TIF) [file pone.0061789.s004.tif]

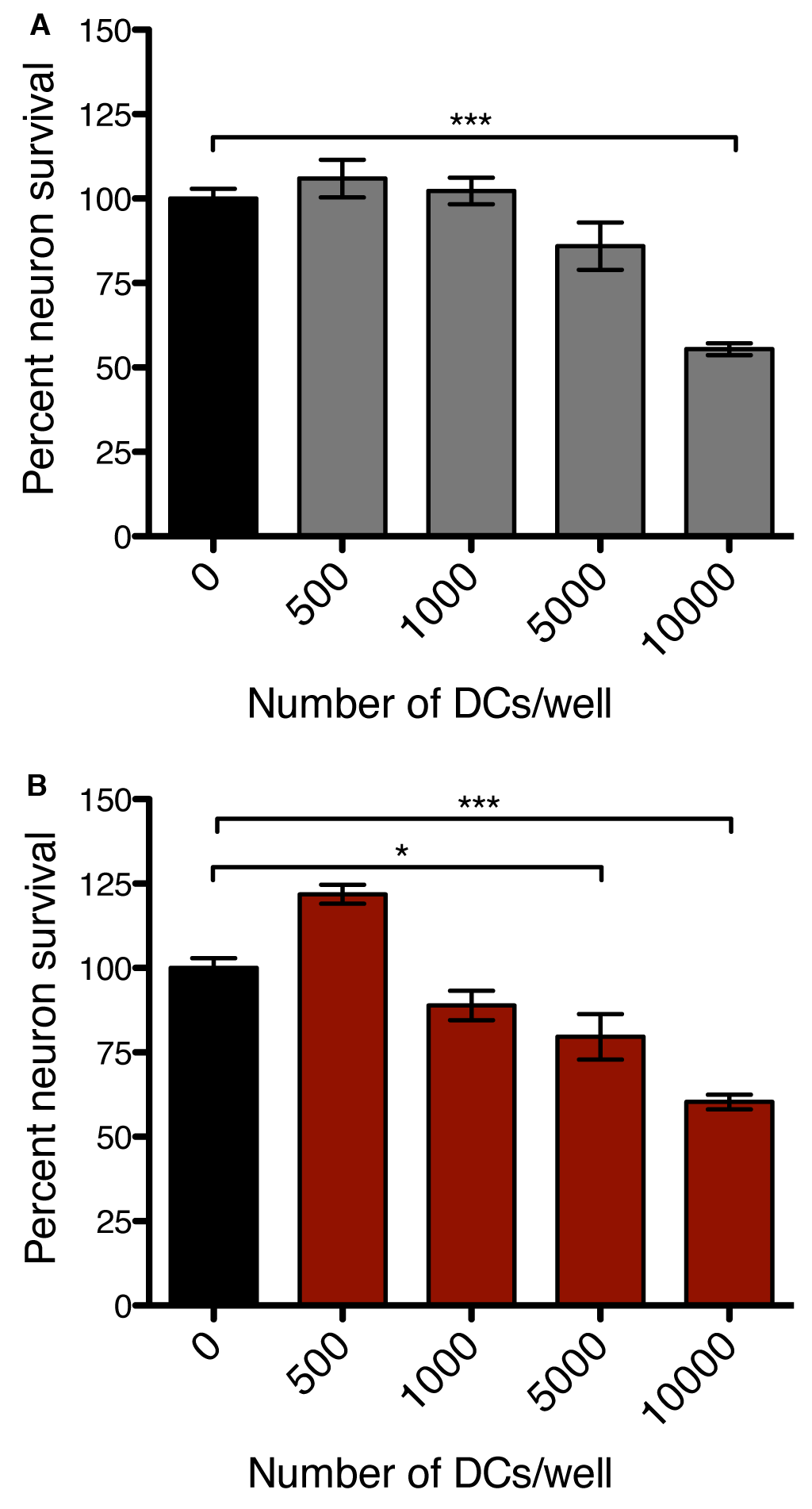

Supplement: Figure S5 — Relative neurotoxicity of transduced DCs in vitro. Quantification of rat primary cortical neuron survival 24 h after exposure to increasing doses of (A) DCs transduced with LV-GFP or (B) DCs transduced with LV-hBDNF. *** P<0.001; * P<0.05 by one-way ANOVA and Tukey post-hoc analysis. n = 48 wells for 0 and 1000; n = 6 wells for 500; n = 18 wells for 5000; n = 12 wells for 10,000. Previously, we found that our rat primary neuron cultures grown in this format contain approximately 10,000–20,000 neurons/well [47] suggesting that in vitro ratios of approximately 1∶40 to 1∶10 transduced DCs:neurons were not neurotoxic, whereas higher ratios of DCs:neurons were neurotoxic. (TIF) [file pone.0061789.s005.tif]

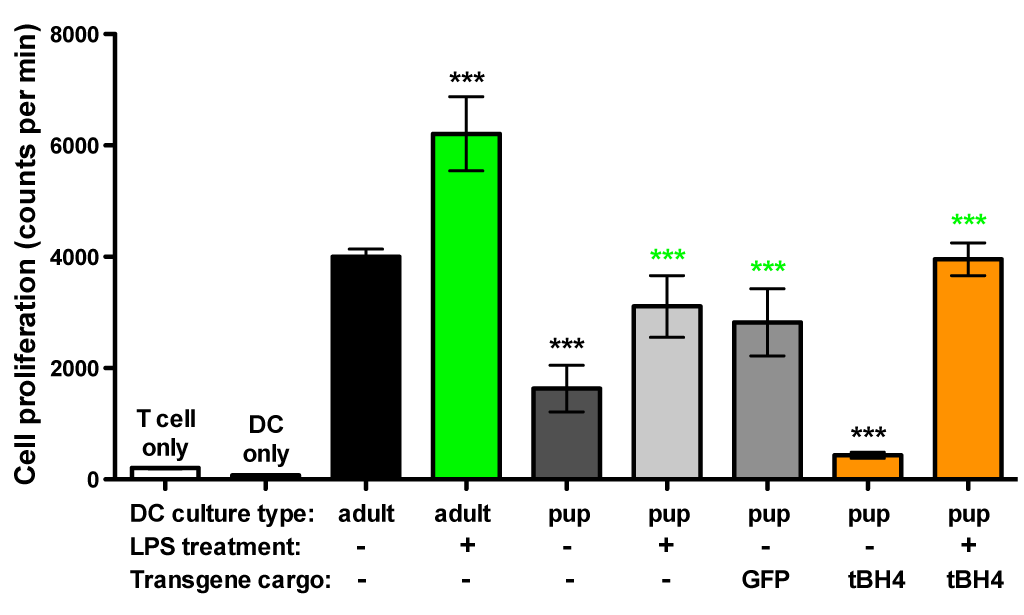

Supplement: Figure S6 — Relative in vitro T cell stimulatory capacity of optimized, transgene loaded-DCs. DCs derived from adult rat bone marrow using standard methods (adult) or from pup bone marrow using the modified method (pup) were tested for their ability to stimulate T cell proliferation in a mixed leukocyte reaction (MLR). LPS-treated DCs were used as a positive control and T cells alone or mitomycin C-treated DCs were used as negative controls. Values represent combined average of wells with DC:T cell ratios of 1∶16 and 1∶8 (6 wells/value). In addition to DC culture type, DC pretreatment with LPS and/or transgene cargo loading is indicated below the x-axis. ***/*** indicates significance by one-way ANOVA plus Tukey post-hoc analysis relative to adult DCs (black asterisks) or relative to adult DCs+LPS (green asterisks). n = 6 wells/group. Error bars denote SEM. (TIF) [file pone.0061789.s006.tif]

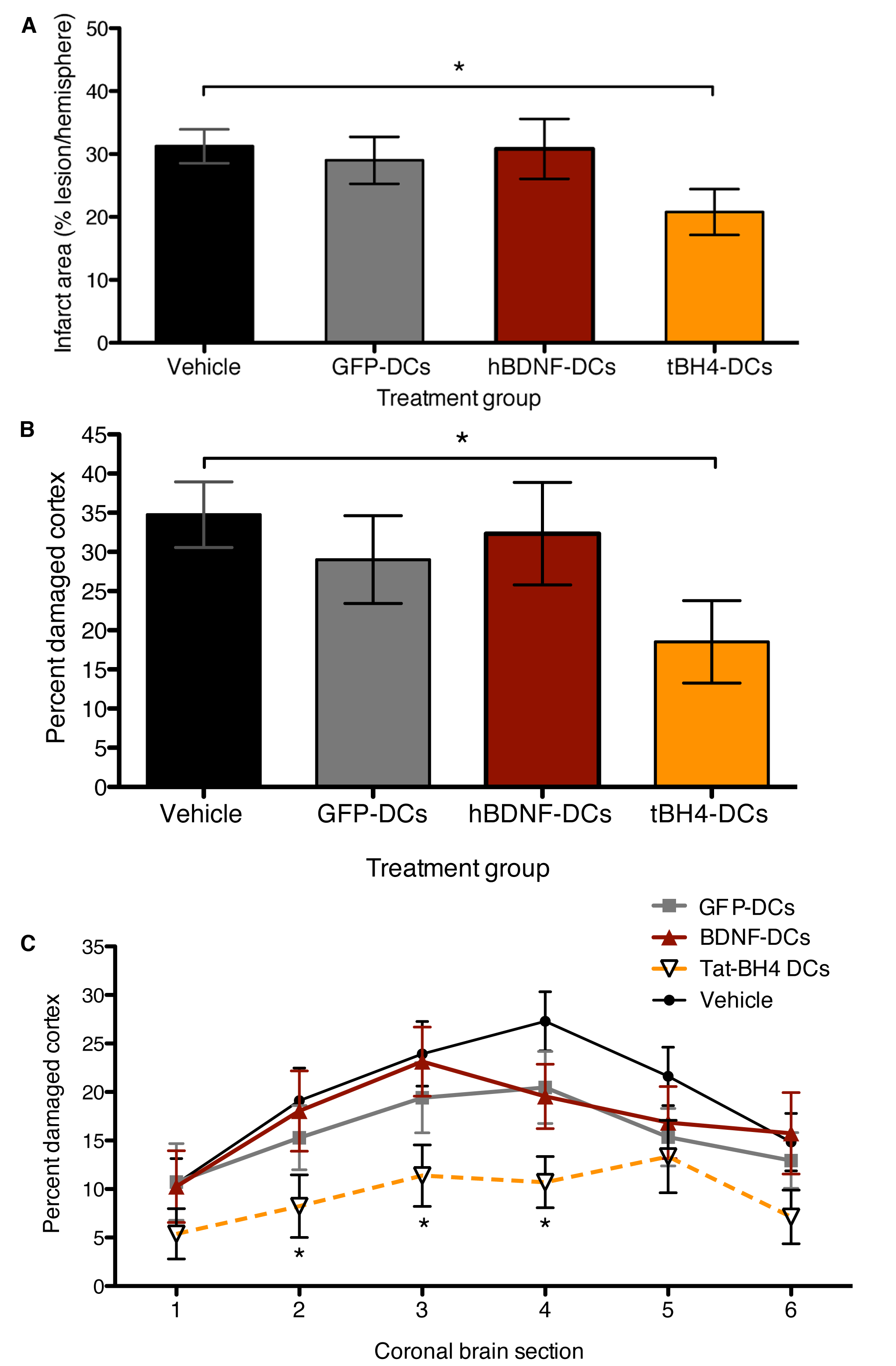

Supplement: Figure S7 — Quantification of ischemic damage following treatment with protein-loaded DCs. (A) Quantification of infarct area for each treatment group, expressed as the percent damage/total hemisphere area. * indicates significance by two-tailed t-test, P = 0.034. Error bars denote SEM. n = 14, 15, 15, 17 for vehicle, GFP-DCs, hBDNF-DCs, and tBH4-DCs, respectively. (B) Quantification of cortical damage for each treatment group at 24 h post-tMCAO, expressed as the percent damage/total cortical area. * indicates significance by two-tailed t-test, P = 0.019. Error bars denote SEM. n = 14, 15, 15, 17 for vehicle, GFP-DCs, hBDNF-DCs, and tBH4-DCs, respectively. (C) Quantification of percent cortical damage at 24 h post-tMCAO by coronal brain section and treatment group. * indicates significance by one-way ANOVA plus Student-Newman-Keuls post-hoc analysis of tBH4-DCs and vehicle treatment for the sum of cortical damage for coronal sections 2–4, P = 0.017. Using the same statistical analysis of cortical damage in coronal sections 2–4 indicated a strong trend towards protection for tBH4-DCs compared to GFP-DC (P = 0.051). Error bars denote SEM. (TIF) [file pone.0061789.s007.tif]

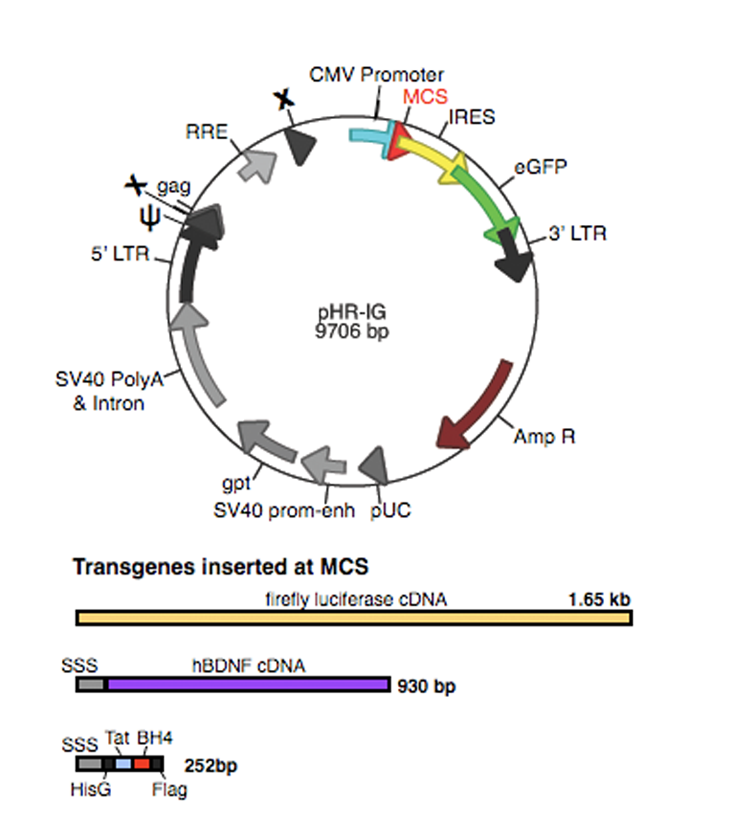

Supplement: Figure S8 — Plasmid map for lentiviral backbone, pHR-IG, and transgene inserts. Plasmid features are indicated with arrows. Abbreviations: CMV = cytomegalovirus; MCS = multiple cloning site; IRES = internal ribosomal entry site; eGFP = enhanced green fluorescent protein; LTR = long terminal repeat; amp R = ampicillin resistance gene; pUC = origin of replication; SV40 prom-enh = simian virus 40 promoter-enhancer; gpt = guanine-hypoxanthine phosphoribosyl transferase; psi = viral packaging sequence; x = 5′ and 3′ splice sites; RRE = rev-responsive element; sss = signal secretory sequence. (TIF) [file pone.0061789.s008.tif]
