# Supplementary material for: Derivation of Injury-Responsive Dendritic Cells for Acute Brain Targeting and Therapeutic Protein Delivery in the Stroke-Injured Rat
Source: PLoS One. 2013 Apr 16;8(4):e61789. doi: 10.1371/journal.pone.0061789 (PMC3627911; doi:10.1371/journal.pone.0061789)
Supplement: Text S1 — Supporting Materials and Methods. (DOC) [file pone.0061789.s009.doc]

**Text S1. Supporting Materials and Methods.**

**Rodent subjects**: All procedures used in this study were approved by the Stanford University Administrative Panel on Laboratory Care and the Association for Assessment of Laboratory Animal Care and are in compliance with the National Institute of Health Guide for the Care and Use of Laboratory Animals. Sprague-Dawley and Fischer F344 rats were obtained from Charles River Laboratories. GFP transgenic rats were received as a gift from G. Steinberg. Subjects were housed in standard conditions with a 12 hour light-dark schedule and fed ad libitum. After 7 days of acclimation, animals were subject to one of the insult models. During all surgeries, animals were maintained at a physiological temperature and respiration rate. Following all surgeries, animals received a subcutaneous injection of the analgesic buprenorphine (0.03 mg/kg).

**Bone marrow-derived DC cultures:** A DC-enriched population was generated from bone marrow as previously described [14,15], with modifications. Briefly, bone marrow from Sprague-Dawley or GFP transgenic rat pups (post-natal day 11 to 17) was isolated and passed through a 70 μm cell strainer. Following red blood cell lysis and 2 cycles of washing with RPMI-1640 (Gibco), cells were resuspended in freezing medium (Gibco) and stored in liquid nitrogen until use (3-9 months). Upon thawing, cells were washed twice with RPMI-1640 (Gibco) and seeded in 6-well plates at a concentration of approximately 2x10^6^ cells/mL in complete medium: RPMI 1640 (Gibco), 10% fetal bovine serum (FBS, Gibco), 2mM L-glutamine (Gibco), 1% nonessential amino acids (Gibco) 1mM sodium pyruvate (Gibco), 0.01% 2-mercaptoethanol (Bio-Rad), 100 μg/mL Gentamicin (Gibco) and the cytokines, interleukin-4, granulocyte-macrophage colony stimulating factor, Flt-3 ligand (5 ng/mL/cytokine, R&D Systems) and incubated at 37°C/5% CO_2_. On the third day in vitro (DIV), each well was given 1 mL complete medium. On DIV 4, medium containing non-adherent cells was removed and replaced with complete medium containing 10 μg/mL protamine sulfate (Sigma), and vehicle or one of the lentivirus (LV) vectors (multiplicity of infection = 10-15). 18 hours post-LV transduction, virus-containing medium was replaced with complete medium (75% new/25% spun-down, conditioned medium from DIV 4. On DIV 5-7, cultures were harvested with PBS and used for in vitro or in vivo experiments. For in vivo experiments, DIV6-7 cultures were used, and harvested DCs were incubated in 50% complete medium/50% RPMI (without cytokines) at 37°C/5% CO_2_ for 1-2 hours prior to in vivo infusion. For lipopolysaccharide (LPS) pre-treatment, LPS-(0.03 μg/mL, Sigma-Aldrich) was added 24 hours prior to harvesting.

**LV vector construction and purification:** Transgene-containing LV particles were generated using a self-inactivating, 3-plasmid system. Transfer vectors containing the transgene, enhanced green fluorescent protein (GFP), with or without a multiple cloning site (MCS) and internal ribosomal entry site (IRES) preceding GFP (pHR-IG and pHR-G, respectively), along with the packaging proteins plasmid (p-delta), and the envelope protein plasmid (encoding vesicular stomatitis virus glycoprotein, VSVG) were a gift from C. Garrison Fathman. Additional transfer vectors were generated with 1 of 3 transgenes inserted in the MCS of pHR-IG (Figure S8). For bioluminescent tracking experiments, cDNA for firefly luciferase (Genbank: X84848) was cloned into pHR-IG using the cut sites, SalI and SacII, to produce the plasmid, pHR-luc-IG. A second plasmid was constructed using cDNA for isoform A exon of human brain-derived neurotrophic factor (hBDNF, Genbank: NM_001143816) received as a gift from R. Tolwani. This was cloned into the SalI/SacII cut sites to produce pHR-hBDNF-IG. A third plasmid was constructed to contain the fusion gene, Tat-BH4 (pHR-tBH4-IG). The Tat-BH4 oligonucleotide was designed to contain a PstI restriction site followed by in-frame signal secretory sequence from rat fibronectin (nt.: 210-305, Genbank: NM_019143.2), a histidine tag (6 histidines + glycine), the tat domain from HIV gag (a.a.: 48-58, Genbank: HM027826.1), the BH4 domain of rat Bcl-xL (a.a. 4-23, Genbank: AF136230), a flag tag (a.a. sequence: MDYKDDDDK) and a stop codon followed by a SacII cut site, and synthesized from 10 overlapping oligos (138-150 bp) by standard oligo synthesis. This product was cloned into pHR-IG to produce pHR-tBH4-IG. Lentiviral particles containing each of these plasmids or pHR-G were produced by standard calcium-phosphate precipitation with human embryonic kidney 293T cells as the packaging cell line as described previously [44], modified for production in 175 cm^2^ flasks. Viral particles were harvested from the culture supernatant 72 hours post-transfection, passed through a 0.45 μm filter and concentrated approximately 100x by ultracentrifugation. Viral pellets were resuspended in RPMI 1640 (GIBCO) containing 5% FBS (GIBCO) and stored at -80°C until use. Vectors made with pHR-G, pHR-hBDNF-IG, pHR-tBH4-IG or pHR-luc-IG were designated LV-GFP, LV-hBDNF, LV-tBH4 and LV-Luc, respectively.

**Flow cytometry:** On DIV 7, bone marrow cultures were harvested in their own medium and washed in FACS buffer (1mM EDTA, 1% FBS in PBS). The following steps were performed at 4°C. For extracellular markers, cells were blocked with 50% FBS in FACS buffer for 10 minutes, washed with FACS buffer, and incubated with primary antibody for 30 minutes on ice (mouse anti-rat Ox42-biotin 1:100 (AbD Serotec), mouse anti-rat CD11c [clone 8A2] 1:20 (AbCam), mouse anti-rat MHC class II RT1B [OX6] 1:50 (AbD Serotec), mouse anti-rat CD80-biotin 1:20 (eBioscience), mouse anti-human CCR2 1:10 (R&D System), mouse anti-rat VLA-4-Alexa Fluor 647 [CD49d] 1:10 (AbD Serotec), mouse IgG1 negative control-Alexa Fluor 647 1:10 (AbD Serotec)). For unconjugated antibodies, cells were washed and incubated with fluorophore-conjugated secondary antibody for 30 minutes (streptavidin-PECy7 1:500 (Invitrogen), goat anti-mouse IgG-RPE [STAR76] 1:10 (AbD Serotec)). Cells were washed again and fixed in 1% paraformaldehyde (PFA) in FACS buffer. For CD68 staining, cells were permeabilized with PFA for 10 minutes, then washed, blocked, washed, and incubated with the primary antibody for 30 minutes (mouse anti-rat CD68-Alexa Fluor 647 1:10 (AbD Serotec)), and fixed in 1% PFA. Cells were run on an LSR II flow cytometer (BDBiosciences) within 5 days. Results were analyzed with FlowJo Software (Tree Star, Inc.).

**In vitro detection of hBDNF and Tat-BH4:** Bone marrow cultures transduced with LV-hBDNF, LV-GFP, or vehicle (complete media + protamine sulfate) were collected at 24, 48, or 72 hours post-transduction, pelleted by centrifugation, and frozen at -80°C. Conditioned medium for each sample was collected by centrifugation and frozen at -80°C. Total protein was extracted from cells by 2 hours incubation in lysis buffer (20mM Tris-HCl, 137mM NaCl, 2mM EDTA, 10% glycerol, 1% Nonidet-P40, plus Complete protease inhibitor (Roche)) at 4°C. Total protein-containing supernatant was collected by centrifugation at 12,000 rpm for 20 minutes at 4°C. Total protein of each sample was quantified using the BCA Protein Assay (Pierce), according to the manufacturer’s microplate protocol. For each sample, hBDNF protein concentration was assayed using the DuoSet hBDNF ELISA kit (R&D Systems), according to the manufacturer’s instructions. The TMB Peroxidase Substrate Kit (Vector Laboratories) was used for final visualization of the antigen-antibody conjugates. For each sample type, protein was collected from 3 separate transductions and run in parallel on a single ELISA plate. Absolute amount of BDNF was calculated based on BDNF protein standards, then normalized to total protein content. For Tat-BH4 detection, LV-GFP- or LV-tBH4-transduced cultures (DIV 7) were fixed with cold methanol and stained with primary antibodies: rabbit-anti-BH4 (1:400, Santa Cruz Biotechnology), mouse-anti-HisG (1:200, Invitrogen), and AlexaFluor-conjugated secondary antibodies (1:400, Invitrogen). Tat-BH4 protein was detected by Western blot (anti-HisG, 1:500, Invitrogen) as described previously [45]. For each assay, protein was collected from 3 separate culture preps.

**Primary neuronal cultures:** Mixed neuronal/glial cortical or hippocampal cultures were prepared from day 18 fetal Sprague-Dawley rats by standard culturing techniques [46,28]. Cells were plated at a density of 1.2 × 10^5^ cells/cm^2^ on 96-well plates coated with poly-D-lysine (Sigma). On DIV 12, cortical cultures were treated with oxygen-glucose deprivation (OGD) and hippocampal cultures were treated with glutamate excitotoxicity. For OGD, media was replaced with 0mM glucose media, and culture plates were put in an anoxic (5% CO2, 95% N2) chamber for 6 hours. After this, culture plates were returned to normoxic conditions and glucose was added back to the media along with vehicle (MEM media, Gibco), GFP-DCs, or hBDNF-DCs (1000 DCs/well). After 18 hours of reperfusion, cultures were fixed with cold methanol and processed to quantify neuron loss. For glutamate excitotoxicity, 50 uM glutamate was added to each well along with vehicle, GFP-DCs, or tBH4-DCs (1000 DCs/well) and plates were fixed with cold methanol 24 hours later. For both assays, DCs were harvested 24 hours prior to addition to neuron cultures and maintained in suspension at a density of 1000 DCs/uL in vehicle at 37°C/5% CO2.

**Quantification of neuron loss:** Primary neuron cultures treated with OGD were analyzed for neuron loss immunohistochemically with a MAP2 antibody, the signal for which was converted to a colorimetric ABTS detection system for quantification [47]. Briefly, culture plates were taken through the following wash/incubation steps: PBS (3x wash), 5% milk/PBS (1 hour, RT), mouse IgG monclonal anti-MAP2/5% milk/PBS (1:400, 30 minutes, RT, Sigma), PBS (3x wash), biotinylated anti-mouse IgG/PBS (1:500, 30 minutes, RT, Vector Laboratories), PBS (3x wash), Vectastain ABC kit (Vector Laboratories), and the ABTS kit (Vector, Burlingame, CA, USA). Optical density at 460 nm was measured on a standard plate reader. Neuron loss was calculated as the percent difference in absorbance for insult/DC-treated wells versus non-insult/non-DC treated and non-insult/DC-treated wells. Values in Figure 4 show neuron loss relative to no insult/DC-treated wells and are from at least 3 independent experiments.

**Mixed leukocyte reaction:** DC cultures derived from freshly-isolated adult bone marrow or cryopreserved, pup bone marrow (postnatal day 15) were generated and transduced with LV vectors and/or treated with LPS as described above. On DIV 7, 5x10^6^ cells per DC population was harvested and inactivated by incubation with mitomycin C (30 μg/mL in 5 mL, Sigma) at 37°C for 30 min. Cells were washed 3 times with RPMI-1640 (Gibco) and plated in a 96-well plate in complete medium (minus cyokines) at 1.25, 2.5, and 5x10^4^ cells/100 μL/well. T-cells were isolated from the spleen of an adult male Sprague-Dawley rat using the Rat T Cell Enrichment Column Kit (RnD Systems) according to the manufacturer’s protocol. Purified T cells were resuspended in complete medium (minus cytokines) and added to DCs at 2x10^5^ T cells/well in an additional 100 μL/well to yield DC:T cell ratios of 1:16, 1:8 and 1:4. Negative control wells contained complete medium alone, 5x10^4^ DCs alone (for each treatment population), or T cells alone. DCs and T cells were co-cultured at 37°C/5% CO2. [^3^H]Thymidine (TdR; 1 μCi) was added to each well for the final 18 hours of the 72 hour co-culture period. Radiolabeled samples were harvested using a multiple well harvester and [^3^H]TdR uptake was determined using a liquid scintillation counter. Each condition (combination of DC type and DC:T cell ratio and negative controls) was tested in triplicate. Relative cell proliferation for each well was calculated by subtracting the average blank value (medium alone) and expressed as counts per minute (cpm). Values obtained with 1:16 and 1:8 DC:T cell ratios were not significantly different and are shown in Supporting Figure S7 as a combined average of all 6 wells/condition.

**Mass spectrometry of standard and modified DC cultures:** DC cultures derived from freshly-isolated adult bone marrow (adult) or cryopreserved, postnatal day 17 pup bone marrow (pup) were generated and transduced with LV-GFP as described above. On DIV 7, 2x10^7^ cells per DC population were harvested and subjected to sub-cellular fractionation by centrifugation. Briefly, cells were lysed in hypotonic buffer with mechanical shearing. The cell lysate was further centrifuged at 10,000 rpm for 1 hour. The cytoplasmic-enriched supernatant fraction was removed from the membrane-enriched pelleted fraction. The enriched membrane-associated fraction was analyzed by LC-MS/MS using a Tempo LC-MALDI Spotting System and the AB Sciex 5800 TOF/TOF Mass Spectrometry System. The resulting spectra were analyzed by ProteinPilot software (v. 3.0, AB Sciex) to identify proteins. Proteins with <2 peptides at 95% coverage were excluded from analysis.

**Transient middle cerebral artery occlusion (tMCAO):** Adult male Sprague-Dawley rats (300-340g) were subject to the proximal model of tMCAO based on previous methods [48], with some modifications. Briefly, using 2.5% isoflurane anesthetic, the left common carotid artery (CCA), external carotid artery (ECA) and internal carotid artery (ICA) were exposed through a midline incision. The ECA was ligated, coagulated, and cut down just proximal to the lingual and maxillary artery branches. Next, a silicon-tipped 4-0 monofilament (Doccol Corporation) was inserted into the ECA and advanced approximately 18 mm into the ICA to occlude the MCA. After 1 hour, the monofilament was withdrawn and the wound closed.

**Intravascular delivery of cultured DCs:** 3 hours post-reperfusion onset, a catheter was inserted into the left ECA and extended towards the ICA. Vehicle (50% RPMI 1640/50% complete media (minus cytokines) or 2x10^6^ LV-transduced cells were delivered as a 0.3 mL infusion at approx. 0.1 mL/minute. Following the infusion, the catheter was removed and the ECA was permanently tied with 5.0 silk suture and the wound closed.

**In vivo tracking of DCs with bioluminescent or SPECT imaging:** Bone marrow-derived DC cultures generated from p17 SD rat pups as described in the corresponding methods section of this manuscript. For bioluminescent imaging, DCs were transduced with the vector, LV-Luc, on DIV 4. On DIV7, DC cultures were harvested in their own media and infused into tMCAO-injured rats 3 hours post-reperfusion. 3 hours later, DC or vehicle-infused rats were anesthetized with 2.5% isoflurane, injected I.P. with 150 mg/kg luciferin dissolved in sterile saline, and imaged for bioluminescence intensity using an IVIS Imaging System at the Stanford Small Animal Imaging Facility. A subset of animals was subjected to a second round of bioluminescent imaging at 24 hours post-DC infusion. For SPECT imaging, DC cultures were harvested in their own media, and radiolabeled according to manufacturer’s recommendations (Ceretec; GE Healthcare; Medi-Physics, Inc., Arlington Heights, IL). In brief, cells for all 4 animals were pelleted, rinsed with saline, pelleted again, and incubated with 10 mCi Tc99m for 15 m at RT. The cells were rinsed, pelleted, and injected via the CCA into each animal (2x10^6^ cells/animal). The radiolabeling efficiency was 800 μCi per 2 million cells. Animals were imaged within 5-20 minutes of injection for a 5 minute exposure on a gamma camera (model 420, Technicare) equipped with a 1 mm pinhole collimator. 10-15 minutes later, animals were imaged on a small-animal SPECT gamma camera (A-SPECT, LumaGEM; Gamma Medica) with the following parameters: 360° rotation, 64 steps, 10 seconds per step, parallel hole collimeter (HRES), 64 × 64 image matrix, and a 7.5 cm field of view. Animals were imaged again on the static gamma camera and SPECT at either 5 or 6 hours post-injection. Percentage of migrated cells in a region of interest (ROI) was determined using ImageQuant TL software (GE Healthcare) by comparing the counts per pixel within an outlined ROI to the counts per pixel from the animal as a whole.

**Permanent MCAO:** Adult male Fischer F344 rats (230-260g) were subject to the distal MCAO model as described previously [49]. Rats were anesthetized with 2.5% isoflurane and the distal MCA was exposed and cauterized permanently above the rhinal fissure. The bilateral common carotid arteries (CCA) were occluded for 1 hour and then released to induce partial reperfusion.

**Perfusion and tissue processing:** To fix brain tissue, animals were transcardially perfused with 0.9% saline followed by 4% paraformaldehyde (PFA). Isolated brains were cryoprotected in 20% sucrose/4% PFA for 24 hours, then snap frozen and stored at -80°C. 30 μm coronal sections were cut on a cryostat, dry-mounted on super frost plus slides (VWR), and re-stored at -80°C until staining. Alternatively, 40 μm sections were cut and stored in freezing media (30% ethylene glycol, 25% glycerol in PBS, pH 7.2-4) at -20°C until staining. For animals subject to the tMCAO model, sets of 6 coronal sections were collected corresponding to the infarct region (anterior/posterior to bregma: +2.5 to -2.0 mm).

**Immunofluorescent staining:** PFA-fixed, dry-mounted sections were used to quantify colocalization of GFP fluorescence with antibodies for GFP, CD11c, and MHC class II at 3 hours post-cell infusion, or the number of CD68-positive cells within the infarct at 24 hours post-reperfusion. Alternatively, free-floating sections were stained with RECA and GFAP to label blood vessels and astrocytes at 10 minutes or 3 hours post-cell infusion. Dry-mounted sections were air-dried for 30 minutes at RT, followed by incubation with proteinase K solution (10 μg/mL, Sigma) for 15 minutes at 37°C. Sections were washed 3x with wash buffer (0.3% triton x-100/PBS) and blocked in 5% goat serum/0.3% triton x-100/PBS for 1 hour at RT. Sections then were incubated with the following primary antibodies: mouse anti-GFP (1:100, Invitrogen), mouse anti-rat MHC class II RT1B (1:50, AbD Serotec), mouse anti-rat CD11c (1:50, AbCam), mouse anti-rat CD68 (1:200, BDPharmingen), anti-RECA-1 (1:200, AbCam), or anti-GFAP (1:200, AbCam) overnight at 4°C. Following 3 washes with wash buffer, sections were incubated with the secondary antibody, goat anti-mouse IgG:Alexa 568 (1/200, Invitrogen) for 1 hour at RT. Sections were washed 2 additional times with wash buffer, nuclei were counterstained with DAPI in PBS (1/1000, Sigma), washed 2 final times, and coverslipped in PVA mounting medium (Sigma). Sections were imaged with an Olympus IX70 microscope and Hamamatsu camera (model C4742-95) powered by a Xenon fluorescent lamp. Using Metamorph Imaging Software (Universal Imaging), GFP fluorescence and its relative colocalization with CD11c and MHC class II staining was imaged at 200x magnification; RECA-1 and CD68 staining were imaged at 200x and 100x magnification, respectively. GFP fluorescence relative to RECA-1 staining also was imaged on a Zeiss 510 meta confocal microscope at 630x magnification. CD11c, MHC class II, and CD68 staining was analyzed with Image J Software (NIH). For CD11c and MHC class II quantification (n = 4/group), 2 blinded observers independently, manually counted the number of cells. For CD68 quantification (n = 8/group), the contrast and brightness was standardized across all images, and a single blinded observer manually counted the number of cells. Parallel sections processed without addition of the primary antibody were essentially blank.

**Infarct quantification:** For quantification of lesion size 24 hours post-reperfusion, PFA-fixed, dry-mounted sections were stained with cresyl violet using the Nissl method. Slides were blinded with respect to treatment group and imaged on a flat-bed scanner. Infarct area was analyzed with ImageJ Software (NIH) by converting the image to black and white, adjusting the brightness and contrast to a standardized level, and outlining the following regions: whole brain slice, infarct hemisphere, and cortex. A standard threshold was set across images and the area above the threshold level of brightness was considered to be damage. Regions such as the ventricles were removed from the damage analysis. The percent damage (area of damage over the total area of the region) in the whole hemisphere, cortex, and striatum was quantified. T-tests were performed to compare total infarct area across 6 representative coronal sections for vehicle-, GFP-DC, tBH4-DC, and hBDNF-DC-infused animals (n-values: vehicle = 14, GFP-DC = 15, hBDNF-DC = 15, tBH4-DC = 17). In addition, a one-way ANOVA was run comparing the infarct sum of coronal sections 2-4 for vehicle-, GFP-DC, and tBH4-DC-injected animals, because this region exhibited the most consistent damage across animals.

**Supplementary References:**

1. Zhang H. *et al.* Hypothermia blocks beta-catenin degradation after focal ischemia in rats. *Brain Res.* **1198**, 182-187 (2008).
